# Structural determination of the complement inhibitory domain of *Borrelia burgdorferi* BBK32 provides insight into classical pathway complement evasion by Lyme disease spirochetes

**DOI:** 10.1371/journal.ppat.1007659

**Published:** 2019-03-21

**Authors:** Jialei Xie, Hui Zhi, Ryan J. Garrigues, Andrew Keightley, Brandon L. Garcia, Jon T. Skare

**Affiliations:** 1 Department of Microbial Pathogenesis and Immunology, College of Medicine, Texas A&M Health Sciences Center, Bryan, Texas, United States of America; 2 Department of Microbiology and Immunology, Brody School of Medicine, East Carolina University, Greenville, North Carolina, United States of America; 3 Division of Molecular Biology and Biochemistry, School of Biological Sciences, University of Missouri-Kansas City, Kansas City, Missouri, United States of America; University of Montana, UNITED STATES

## Abstract

The carboxy-terminal domain of the BBK32 protein from *Borrelia burgdorferi sensu stricto*, termed BBK32-C, binds and inhibits the initiating serine protease of the human classical complement pathway, C1r. In this study we investigated the function of BBK32 orthologues of the Lyme-associated *Borrelia burgdorferi sensu lato* complex, designated BAD16 from *B*. *afzelii* strain PGau and BGD19 from *B*. *garinii* strain IP90. Our data show that *B*. *afzelii* BAD16-C exhibits BBK32-C-like activities in all assays tested, including high-affinity binding to purified C1r protease and C1 complex, and potent inhibition of the classical complement pathway. Recombinant *B*. *garinii* BGD19-C also bound C1 and C1r with high-affinity yet exhibited significantly reduced *in vitro* complement inhibitory activities relative to BBK32-C or BAD16-C. Interestingly, natively produced BGD19 weakly recognized C1r relative to BBK32 and BAD16 and, unlike these proteins, BGD19 did not confer significant protection from serum killing. Site-directed mutagenesis was performed to convert BBK32-C to resemble BGD19-C at three residue positions that are identical between BBK32 and BAD16 but different in BGD19. The resulting chimeric protein was designated BXK32-C and this BBK32-C variant mimicked the properties observed for BGD19-C. To query the disparate complement inhibitory activities of BBK32 orthologues, the crystal structure of BBK32-C was solved to 1.7Å limiting resolution. BBK32-C adopts an anti-parallel four-helix bundle fold with a fifth alpha-helix protruding from the helical core. The structure revealed that the three residues targeted in the BXK32-C chimera are surface-exposed, further supporting their potential relevance in C1r binding and inhibition. Additional binding assays showed that BBK32-C only recognized C1r fragments containing the serine protease domain. The structure-function studies reported here improve our understanding of how BBK32 recognizes and inhibits C1r and provide new insight into complement evasion mechanisms of Lyme-associated spirochetes of the *B*. *burgdorferi sensu lato* complex.

## Author summary

Lyme disease, caused by *Borrelia burgdorferi sensu lato* complex, is the most common tick-borne infection in the Northern hemisphere. As a pathogen that is transmitted within blood and body fluids, *Borrelia* species must combat the complement system, which is a soluble host defense pathway that marks invaders for elimination. Previously, we demonstrated that the *B*. *burgdorferi* protein BBK32 blocks the activation of the classical pathway of complement by binding to and inhibiting the initiating protease, C1r. Here we show that the BBK32 orthologue from *B*. *garinii*, designated BGD19, is unable to neutralize complement-mediated serum killing. In contrast, BBK32 and the *B*. *afzelii* orthologue BAD16 potently inhibits serum killing. Comparison of the amino acid sequences of these proteins identified three residues unique to BGD19 that contribute to its reduced inhibitory activity. The three-dimensional structure of BBK32-C, reported here, showed that these residue positions are surface exposed in BBK32, further supporting their role in C1r inhibition. These results render important insight into how Lyme disease-associated spirochetes recognize and block an important human innate immune pathway and provide mechanistic insight into evolutionarily optimized C1r inhibitors.

## Introduction

Spirochetes belonging to the *Borrelia burgdorferi sensu lato* complex are the causative agent of Lyme borreliosis and include *B*. *burgdorferi sensu stricto*, *B*. *garinii*, and *B*. *afzelii*. *B*. *burgdorferi sensu stricto* (referred to as *B*. *burgdorferi* hereafter) causes 300,000 cases of Lyme disease in the United States each year [1], while *B*. *garinii* and *B*. *afzelii* are the most common etiological agent of Lyme disease in Europe and Asia [2]. *B*. *burgdorferi sensu lato* are the leading arthropod-borne infectious agents in the Northern hemisphere and are capable of hematogenous dissemination whereby a wide range of remote host tissues are colonized. To survive and persist in immunocompetent hosts, Lyme-associated spirochetes must evade host immune defenses including the evolutionarily ancient proteolytic cascade of innate immunity known as the complement system.

Complement is a group of nearly three dozen proteins that combine to coordinate a tightly controlled set of proteolytic reactions directed at target cell surfaces [3,4]. Complement activation is initiated by soluble pattern recognition proteins which are capable of discerning foreign molecular surfaces. The specific mode of recognition defines the three conventional pathways of complement known as the classical pathway, lectin pathway, and alternative pathway. For instance, the classical pathway is activated upon binding of the complement protein C1q to antigen-bound antibodies (i.e. immune complexes). Likewise, the lectin pathway is activated following the binding of mannose-binding lectin or ficolins to foreign carbohydrate structures, while the alternative pathway is constitutively activated at low levels via a mechanism referred to as ‘tick-over’ [3,4]. Independent of the molecular initiating event, all three pathways proceed by activation of a series of specialized serine proteases that converge on the central molecule of complement, C3. C3 is cleaved by enzymatic complexes called C3 convertases which results in complement amplification at the target surface, activation of the terminal pathway of complement, and induction of downstream effector functions. Complement activation ultimately results in the opsonization of targeted surfaces, recruitment of professional phagocytes, and direct lysis of susceptible membranes [3,4].

Host cells express several proteins which function to regulate complement and thus are typically protected from unintended targeting by the cascade. In contrast, pathogens that traffic in fluids where complement is present at high concentrations have necessarily evolved mechanisms to evade complement detection and activation. For example, many human pathogens secrete membrane-associated proteins which bind endogenous host regulators of complement [5]. In this regard, a prominent bacterial target is the dominant negative regulator of the alternative pathway, factor H. Indeed, *B*. *burgdorferi sensu lato* species themselves are known to encode up to five distinct proteins (CspA, CspZ, ErpP, ErpC, and ErpA; note that the latter three are also referred to OspE-related proteins; Erps) that bind to and recruit factor H to the bacterial surface, thereby hijacking its complement protective activities [6–13]. In addition to factor H-binding proteins, distinct borrelial proteins are known that specifically block the formation of the membrane attack complex [14–16], recruit host plasminogen and degrade complement components [17–20], or bind directly to other complement components [21,22]. In total nearly a dozen *B*. *burgdorferi sensu lato* proteins have now been identified that exhibit specific complement inhibitory activities [23].

Within the small arsenal of borrelial complement inhibitors, the surface-expressed lipoprotein *B*. *burgdorferi* BBK32 remains the lone identified and characterized classical pathway-specific inhibitor [22]. The classical pathway is controlled by the action of the first component of complement, C1, which is a multi-protein complex composed of C1q bound to a heterotetramer of two serine proteases, C1r and C1s (**S1 Fig**). C1 thereby functions as both the pattern recognition molecule and initiating zymogen of the classical pathway. The C1 complex circulates in blood in an inactive form until C1q is recruited to the surface via recognition of receptors such as immune complexes. C1q-binding promotes autocatalytic activation of the C1r protease within the C1 complex which then cleaves C1s to form fully activated C1. At this step the C1s enzyme cleaves complement components C2 and C4, and the classical pathway intersects the lectin pathway at the formation of classical/lectin pathway C3 convertases (C4b2a). C3 convertases then convert complement C3 into its activated forms, which in turn drive downstream reactions of the cascade. Previously we have shown that the C-terminal globular domain of *B*. *burgdorferi* BBK32 (termed BBK32-C) blocks classical pathway activation by binding with high-affinity to the initiating serine protease C1r, and preventing both its autocatalytic and C1s cleavage activities within the C1 complex [22] (**S1 Fig)**.

In this study we investigated the activity of BBK32 orthologues encoded by the prevalent *B*. *burgdorferi sensu lato* species *B*. *garinii* and *B*. *afzelii* to better understand the structural and mechanistic basis for BBK32-mediated C1r inhibition. Herein we report the first crystal structure of the anti-complement domain of BBK32 which reveals a novel helical bundle fold. A chimeric mutagenesis strategy provided additional insight into the reduced *in vitro* activities of *B*. *garinii* BBK32 orthologue BGD19 relative to the *B*. *afzelii* BBK32 orthologue BAD16 and *B*. *burgdorferi* BBK32 itself. Biochemical studies were then used to map the BBK32 binding site on human C1r and demonstrated that the serine protease (SP) domain was required for BBK32/C1r complex formation. The results of this study significantly improve our understanding of the unique classical pathway inhibition properties of BBK32 and suggest that BBK32-mediated complement evasion activity is shared across major species of Lyme disease-associated spirochetes.

## Results

### BBK32 orthologues identified within *Borrelia burgdorferi sensu lato* complex

**Fig 1:**
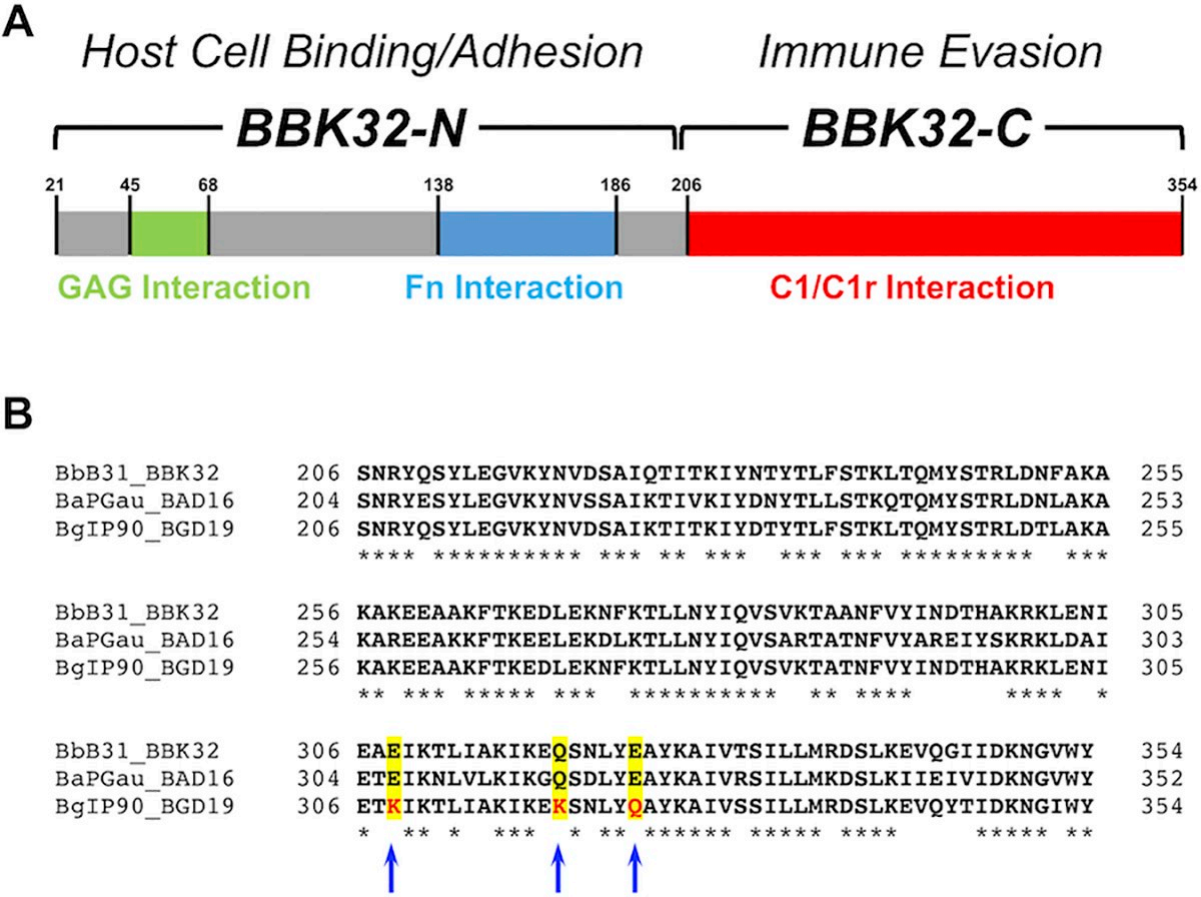
BBK32 orthologues are encoded by *Borrelia burgdorferi sensu lato* isolates. A) BBK32 is a multifunctional lipoprotein expressed on the surface of *B*. *burgdorferi*. BBK32 interacts with three vertebrate host macromolecules via non-overlapping binding sites. The intrinsically disordered N-terminal domain of BBK32 (BBK32-N) recognizes certain glycosaminoglycans and the human extracellular matrix protein fibronectin, while the globular C-terminal region (BBK32-C) binds to the complement protease C1r within the C1 complex. B) A sequence alignment of the C-terminal domain of BBK32 orthologues from the Lyme disease-associated spirochetes *B*. *burgdorferi* BBK32 from strain B31, *B*. *garinii* BGD19 from strain IP90, and *B*. *afzelii* BAD16 from strain PGau is shown. Residues selected for mutational analysis in this study (i.e. “BXK32-C”) are highlighted in yellow and marked with arrows.

*B*. *burgdorferi* BBK32 is a surface-exposed lipoprotein with multiple roles in vertebrate host interactions [22,24–32]. Nearly two decades ago BBK32 was shown to interact with human fibronectin [29] and later with certain host glycosaminoglycans [28]. Owing to these activities BBK32 has long been viewed as the prototypical *B*. *burgdorferi* adhesin and has been shown to play critical roles in colonization and dissemination [24–26,33]. While the interaction of BBK32 with fibronectin and glycosaminoglycans is formed by non-overlapping binding sites in the intrinsically disordered N-terminal region of BBK32 (BBK32-N) [28,30,31,34,35], the globular C-terminal region of BBK32 (BBK32-C) is now known to bind specifically to the initiating protease of the classical complement pathway, C1r [22] (**Fig 1A**). Orthologues to *B*. *burgdorferi* BBK32 within the *sensu lato* complex are encoded by *B*. *afzelii* (termed BAD16) and *B*. *garinii* (termed BGD19). These BBK32 orthologues share ~90% and ~70% overall amino acid sequence identity relative to *B*. *burgdorferi* BBK32, respectively (**Fig 1B**).

### Recombinant BBK32 orthologues from *B*. *garinii* and *B*. *afzelii* bind with high affinity to C1 and C1r

**Fig 2:**
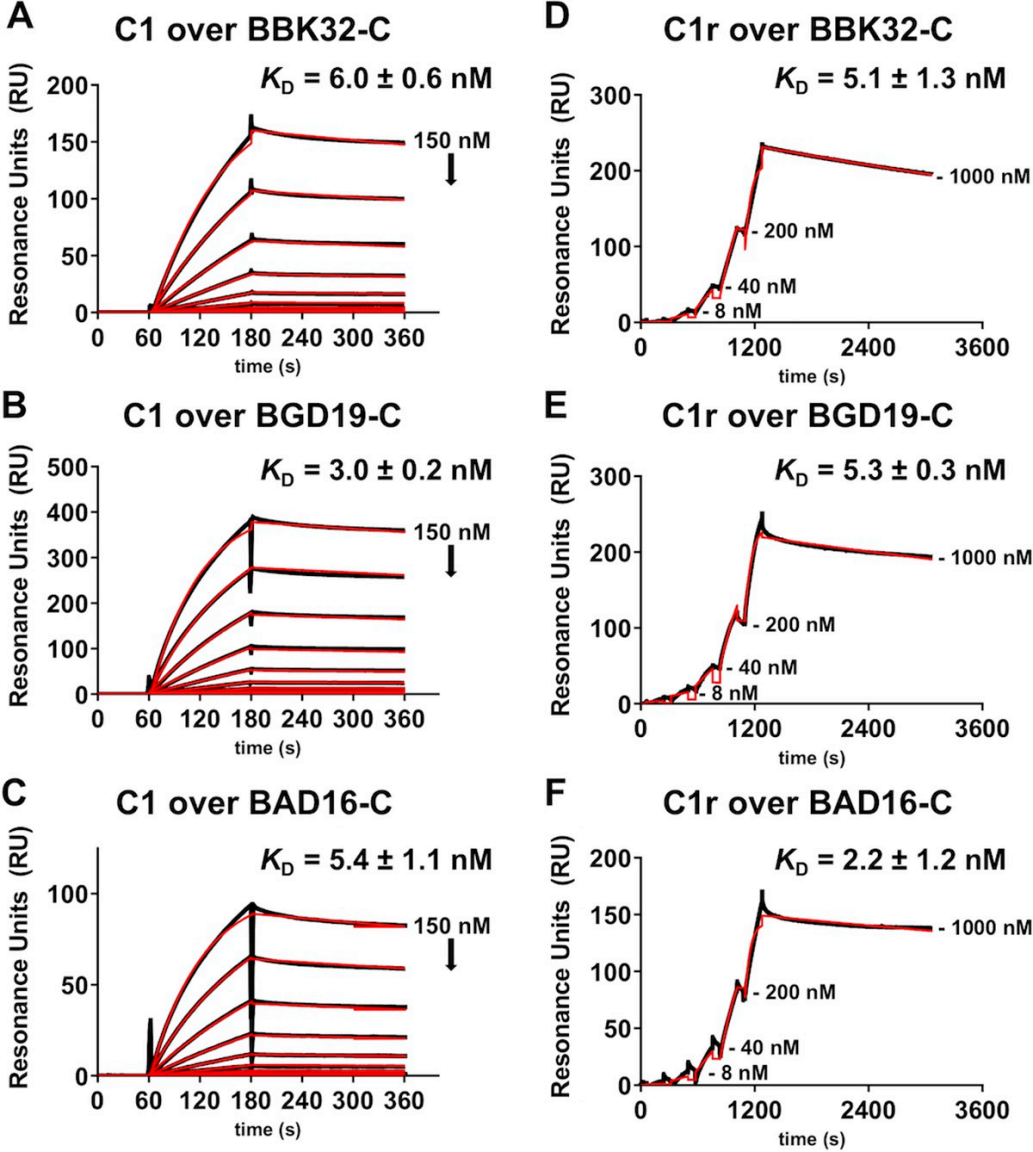
The C-terminal domain of BGD19 and BAD16 bind with high affinity to human C1 and C1r. The ability of the C-terminal region of BGD19 (BGD19-C) and BAD16 (BAD16-C) to bind human C1 or C1r, was assessed by SPR. BBK32-C was used as a control. For C1, a two-fold dilution series (0.1 to 150 nM) was injected over immobilized BBK32-C (panel A), BGD19-C (panel B), and BAD16-C (panel C). The raw sensorgrams are drawn as black lines and the results of kinetic fitting analysis using Biacore T200 Evaluation Software are drawn as red lines. For C1r the a single-cycle analysis was performed using a five-fold dilution series (1.6 to 1000 nM) of BBK32-C (panel D), BGD19-C (panel E), and BAD16-C (panel F). For clarity, the dissociation phase of each sensorgram is labeled with the C1r injection concentration. Sensorgrams from a representative injection series are shown and all experiments were conducted in triplicate with the dissociation constants (*K*_D_) reported as the mean ± S.D.

Among the *B*. *burgdorferi sensu lato* complex *in vitro* studies have shown that *B*. *garinii* is more sensitive to the lytic component of human serum (i.e. the complement membrane attack complex) than *B*. *burgdorferi* or *B*. *afzelii* [36]. Increased alternative pathway complement activity has been posited as an explanation, as *B*. *garinii* does not recruit functional human factor H via factor H-binding proteins, unlike human serum-resistant *B*. *burgdorferi* and *B*. *afzelii* [37]. However, prior work showed that *B*. *garinii* strains are also human serum sensitive in a C1q-dependent manner, indicating a potential role for the classical complement pathway in the human serum susceptibility phenotype of *B*. *garinii* [36]. In this regard the relative classical pathway-specific complement inhibitory activities of the BBK32 orthologues *B*. *garinii* BGD19 and *B*. *afzelii* BAD16 are unknown. To address this, we produced recombinant proteins of each orthologue corresponding to the C-terminal complement inhibitory region of BBK32 (i.e. BBK32-C) (**Fig 1B**), and evaluated their ability to bind to human C1 complex and isolated human C1r using surface plasmon resonance (SPR). These experiments show that BAD16-C and BGD19-C each bind to human C1 and C1r with high affinity (**Fig 2, S1 Table**).

### *B*. *garinii* BGD19 has significantly reduced complement inhibitory activity relative to *B*. *burgdorferi* BBK32 or *B*. *afzelii* BAD16

**Fig 3:**
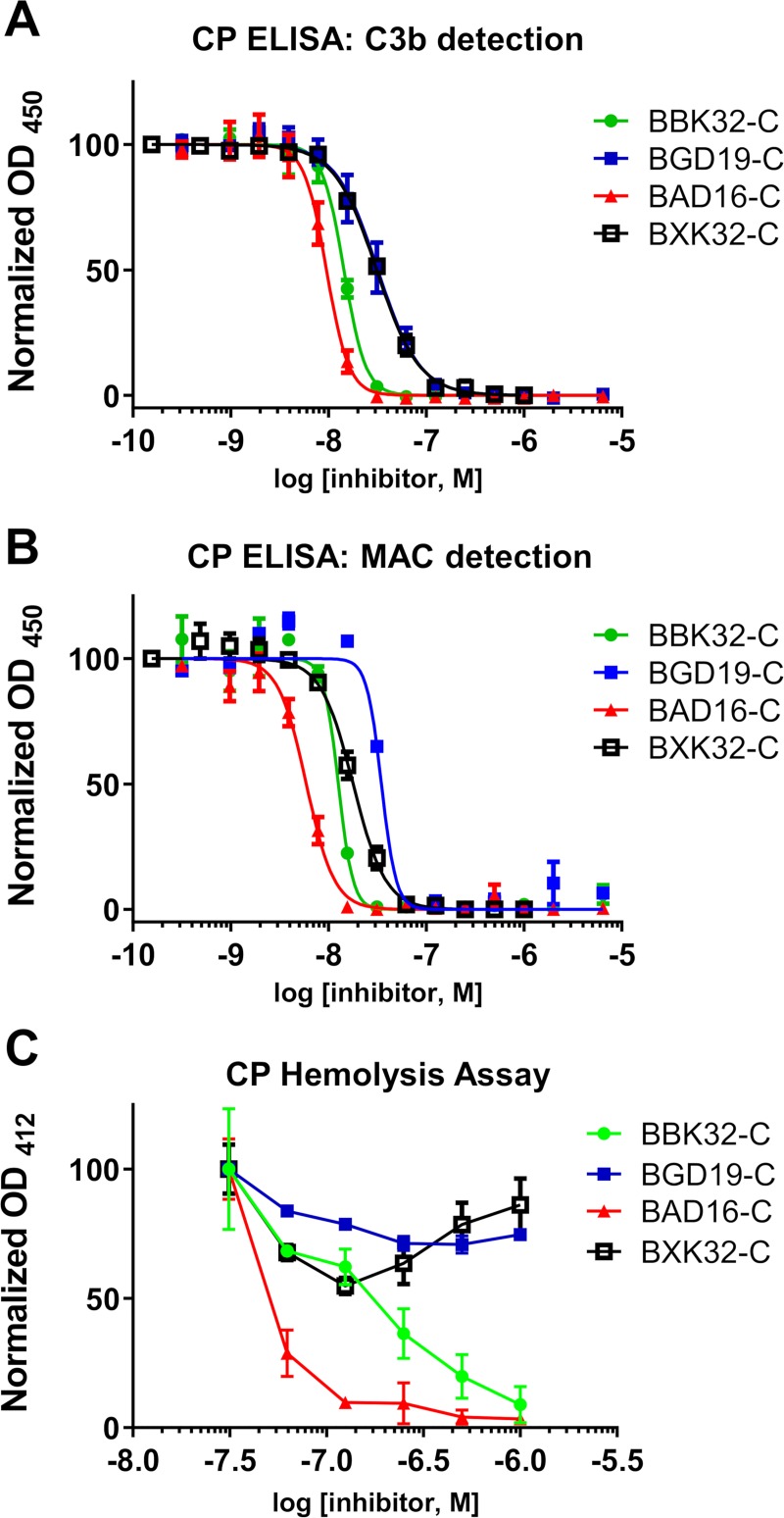
BGD19 and BAD16 inhibit the classical pathway of complement. A) Two *in vitro* assays of classical pathway complement activation were used to assess the relative inhibitory activity of recombinant BBK32-C, BGD19-C, BAD16-C, and BXK32-C. (A-B) an ELISA-based assay was used in the presence of a two-fold concentration series of BBK32-C, BGD19-C, BAD16-C, and BXK32-C (1 nM to 2,000 nM). A) C3b deposition or B) MAC deposition was detected in separate experiments each performed in duplicate. C) A classical pathway-specific hemolytic assay was used in the presence of a concentration series of each inhibitor (31 to 1,000 nM) to assess the relative ability of each protein to protect sensitized sheep red blood cells from complement-mediated lysis in 1% normal human serum. Each experiment was performed in triplicate and values are reported as the mean ± SEM.

Previously we have shown that the high-affinity interaction formed between BBK32-C and human C1r correlates with blockade of classical pathway activation in assays where human serum is used as the source of complement [22]. In a classical pathway-specific ELISA assay that monitors the deposition of the complement activation products C3b or membrane attack complex (MAC) we found that BGD19-C (IC_50, C3b deposition_ = 32 nM; IC_50, MAC deposition_ = 35 nM) exhibited a two to three fold decrease in potency relative to BBK32-C (IC_50, C3b deposition_ = 14 nM; IC_50, MAC deposition_ = 13 nM), whereas BAD16-C exhibited no greater than a two-fold increase in potency (IC_50, C3b deposition_ = 9.6 nM; IC_50, MAC deposition_ = 5.9 nM) (**Fig 3A and 3B**) based on non-overlapping 95% confidence intervals (**S2 Table**). In a classical pathway hemolysis assay we found a larger difference in relative potency as concentrations of *B*. *garinii* BGD19 up to 1 μM failed to exhibit saturable inhibition of classical pathway activation in hemolysis assays (estimated IC_50_ ~ 7,600 nM), unlike BBK32 (IC_50_ = 170 nM) or BAD16 IC_50_ = 55 nM) which both exhibit dose-dependent protection of sheep red blood cell lysis by human serum (**Fig 3C, S2 Table**).

Sequence alignment of BBK32, BAD16, and BGD19 reveals there are just three non-conservatively substituted amino acids shared between BBK32-C and BAD16-C that are different in BGD19-C (see highlighted residues and arrows **Fig 1B**). In *B*. *burgdorferi* BBK32 and *B*. *afzelii* BAD16 these residues are Glu-308, Gln-319, and Glu-324, whereas in *B*. *garinii* BGD19 these positions are changed to Lys-308, Lys-319, and Gln-324. To investigate the potential role of these residues in mediating C1r inhibition, we produced a chimeric BBK32-C protein, termed BXK32-C, where each residue was changed to the *B*. *garinii* BGD19 residue (i.e. BBK32-E308K-Q319K-E324Q). Interestingly, the inhibitory activity of the chimeric BXK32-C shifts from that of BBK32 to BGD19 (**Fig 3A–3C**). These data indicate that residues encoded at one or more of these positions in BGD19 likely contribute to its observed reduction in human classical complement pathway inhibitory activity.

**Fig 4:**
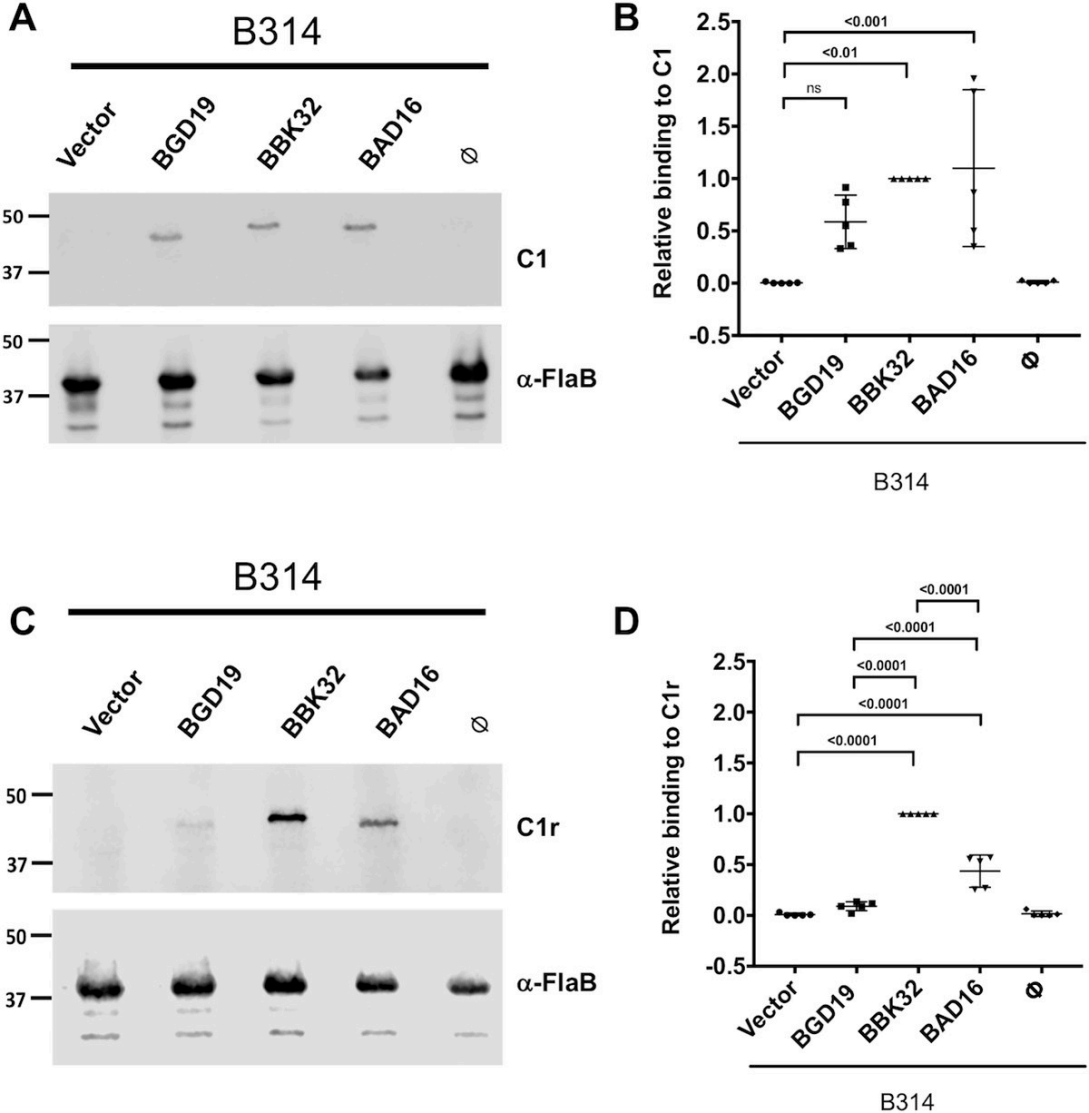
Binding of C1 and C1r to BBK32 orthologues via far western blot analysis. A-D) BGD19, BBK32, and BAD16 were expressed as lipoproteins on the surface of *B*. *burgdorferi* B314. Whole cell protein lysates were separated on an SDS-PAGE gel and probed for binding to human C1 (panel A) or C1r (panel C) using a Far Western blot overlay. Samples tested include strain B314/pBBE22*luc* (vector only control; labeled as “Vector”), B314/pBDG19 (labeled as BGD19), B314/pCD100 (labeled as BBK32), B314/pBAD16 (labeled as BAD16), and B314 alone (labeled as null). FlaB was used as a loading control to normalize variation between C1 and C1r binding by BBK32, BAD16, and BGD19 in panels A and C. Densitometry was performed from independent blots to quantify the observed signals as depicted in panels A and C. Panels B and D report the signal detected for C1 and C1r to the samples indicated on the x axis, respectively. All values were normalized relative to BBK32 binding to either C1 or C1r. P values between samples are indicated above the bars.

Next, we investigated the activity of BGD19 and BAD16 when expressed as full-length lipoproteins on the spirochetal surface by using the poorly adherent, non-infectious strain B314 of *B*. *burgdorferi*. A shuttle vector containing each orthologous *bbk32* gene controlled by its native promoter was constructed, transformed into strain B314, and designated as B314/pCD100 (*B*. *burgdorferi bbk32*) [22], B314/pBGD19 (*B*. *garinii bgd19*), and B314/pBAD16 (*B*. *afzelii bad16*) (**S2A and S2B Fig**). BBK32, BGD19 and BAD16 were expressed heterologously in *B*. *burgdorferi* strain B314 and surface localization was assessed using the proteinase K accessibility assay. Here, BAD16 and BGD19 were sensitive to protease digestion under conditions that the subsurface endoflagellar protein, FlaB, was not affected, indicating that the BBK32 orthologues were surface exposed and that the borrelial cells were structurally intact, respectively (**S3 Fig**). Additionally, we assessed the different transcript levels of the *bbk32* orthologues by qRT-PCR analysis and found that all orthologues were expressed at levels that were not significantly different (**S2C Fig**). Next, Far Western blot experiments were performed using biotinylated-human C1 (**Fig 4A and 4B**) or human C1r (**Fig 4C and 4D**) as probes and normalized to the levels of *B*. *burgdorferi* BBK32 produced. These data suggest that BGD19 and BAD16 bind with similar affinity to human C1 (**Fig 4B**). However, when C1r is used as the probe, BGD19 binds only weakly and not significantly different relative to a vector only control (denoted as “Vector”), whereas BBK32 and BAD16 each bind to C1r (**Fig 4D**).

**Fig 5:**
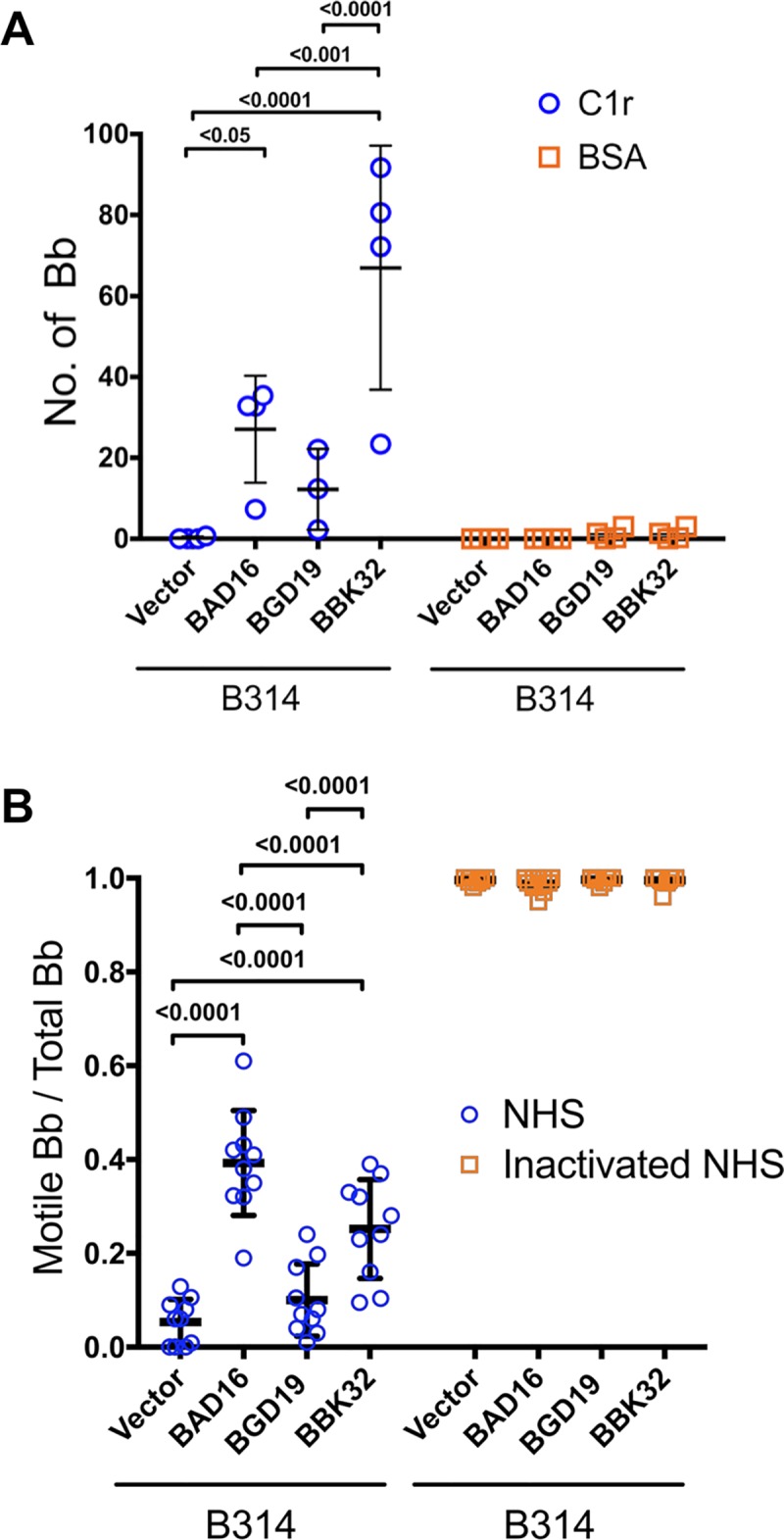
Native BAD16 and BGD19 exhibit differential binding to C1 and C1r and confer serum-resistance when expressed on the surface of spirochetes. A) Natively expressed *bad16* (labeled as BAD16) and *bgd19* (labeled as BGD19), were tested for their ability to bind immobilized C1r (blue circles) or BSA (yellow squares) relative to B314 containing BBK32 (labeled as BBK32) or B314 with vector DNA alone (labeled as Vector). Binding was done in triplicate for independent samples and the average and standard deviation shown. B) The ability of each protein to confer resistance to normal human serum (NHS) was assessed in the serum sensitive *B*. *burgdorferi* strain B314. Sensitivity was scored as a ratio of the affected cells relative to the total cells viewed. Cells affected were categorized as those that lacked motility, exhibited membrane damage, or manifested overt cell lysis (blue circles). Heat inactivated NHS was used as a control and is shown on the right (yellow squares). P values between samples are indicated above the bars. ns, not significant.

To assess the binding of C1r to native, surface exposed BAD16 and BGD19 relative to BBK32, we incubated whole, intact borrelial strain B314 cells with immobilized C1r. In agreement with the whole cell lysate assays (**Fig 4**) BGD19 showed significantly reduced C1r binding relative to BBK32 and BAD16 (**Fig 5A**). Next, we assessed the ability of heterologously expressed BGD19 and BAD16 to confer serum resistance to strain B314. While expression of BBK32 and BAD16 at the B314 surface protected spirochetes from classical pathway-mediated complement killing, BGD19 did not significantly reduce killing relative to the vector only control (**Fig 5B**). Interestingly, BAD16 exhibited greater resistance to serum relative to BBK32 (**Fig 5B**).

### The crystal structure of *B*. *burgdorferi* BBK32-C determined to 1.7Å resolution

Circular dichroism studies indicate that the secondary structure of BBK32-C is predominantly helical in solution [38]. Beyond this, little is known about the structure of the complement inhibitory domain of BBK32. To address this, we initiated crystallographic studies with the goal of determining a high-resolution structure of BBK32-C. Attempts to crystallize the original BBK32-C construct (i.e. residues 206–354) were unsuccessful. Preliminary limited proteolysis experiments suggested that flexible residues were present at the N- and C-termini in BBK32-C. A number of constructs were designed to truncate BBK32, ultimately yielding a C-terminal truncation mutant lacking six residues (i.e. BBK32_(206–348)_) which produced protein crystals (see Methods and Materials). Importantly, the BBK32_(206–348)_ construct retained full C1-binding, C1r-binding and complement inhibitory activities (**S4A–S4C Fig**).

**Fig 6:**
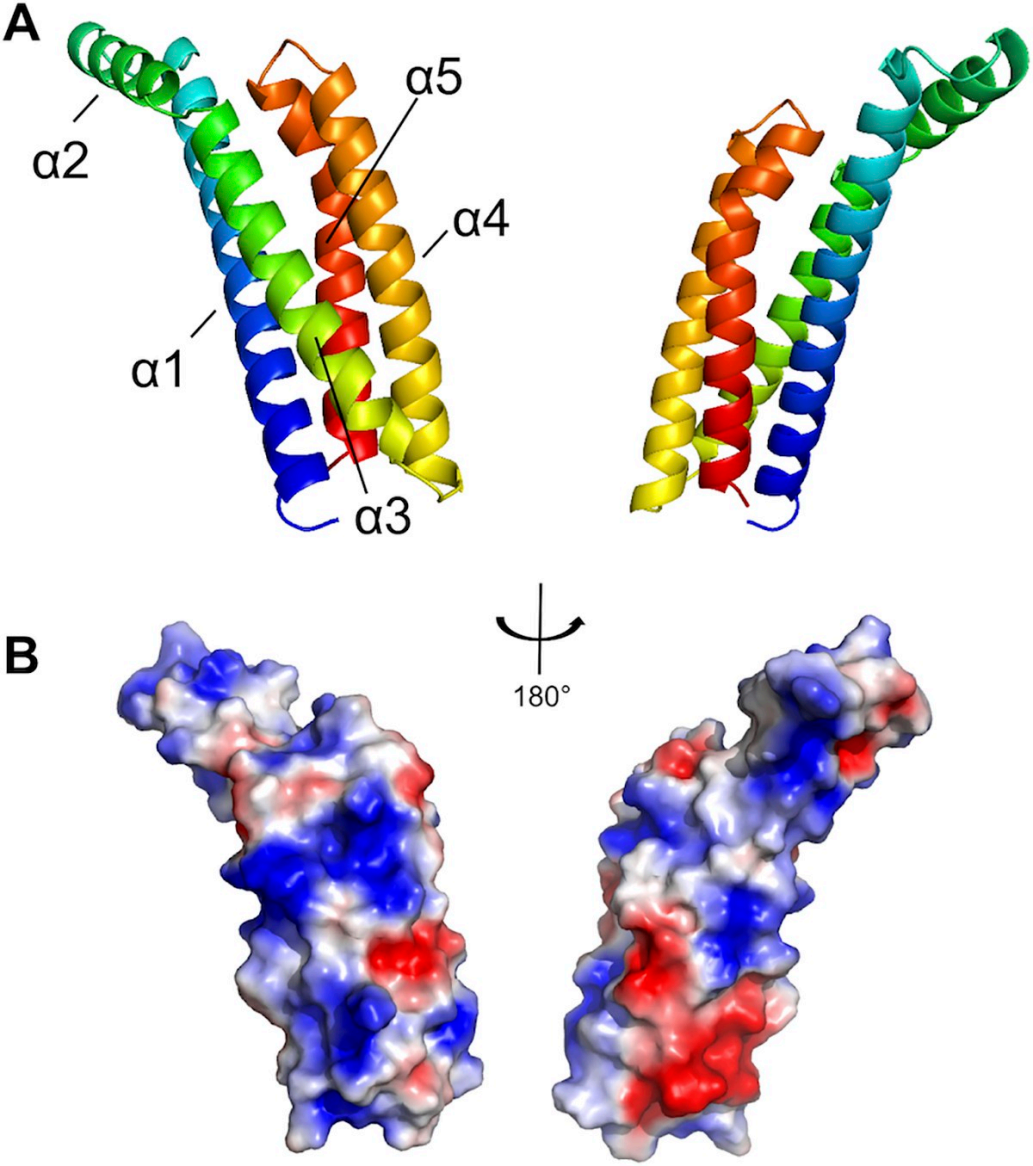
The crystal structure of the complement inhibitory domain of BBK32. A) The structure of BBK32_(206–348)_ solved at 1.7Å resolution (PDB: 6N1L). A ribbon diagram representation using a spectrum-based coloration scheme of BBK32_(206–348)_ where the N-terminal region of the protein is colored in blue and the C-terminus in red. The structure is shown turned 180° about the y-axis. BBK32_(206–348)_ is characterized by a helical bundle fold where helices 1, 3, 4, and 5 form a core four-helix bundle motif and helix 2 extends away from the core at ~120° relative to helix 3. B) BBK32 is drawn in a surface representation in the same orientations as depicted in panel A. The Adaptive Poisson-Boltzmann Solver as implemented in Pymol was used to calculate the electrostatic potential of the molecular surface. The color scheme represents a gradient of electrostatic potential where regions of negative (red) and positive (blue) are contoured at ± 2 k_b_T/e where k_b_ is Boltzmann’s constant = 1.3806 x 10^−23^ J K^-1^, T is temperature in K, and e is the charge of an electron = 1.6022 x10^-19^ C.

BBK32_(206–348)_ crystals grew in space group *P*6_5_ with one molecule per asymmetric unit and diffracted to 1.7 Å resolution (**Table 1**). BBK32_(206–348)_ consists of five α-helices and adopts a helical bundle fold (**Fig 6A, S4D Fig**). Starting from the N-terminus, helix α1 (residues Ser-211 to Met-245) interacts with helices α3 (Lys- 256 to Ala-286), α4 (Ile-293 to Lys-317), and α5 (Leu-323 to Ile-347) to form an anti-parallel four-helix bundle. Helix α2 (Asn-251 to Ala-261) does not participate in the core bundle motif but rather forms hydrophobic interactions with helix α1 and sits at an angle of ~120° relative to helix 3. Adaptive Poisson-Boltzmann Solver software was used to calculate the electrostatic potential of the BBK32-C molecular surface [39] (**Fig 6B**). The protein surface is characterized by several contiguous positively charged regions with a larger negatively charged surface being formed where the C-terminal and N-terminal helices meet. Overall the structure of BBK32-C is best characterized by a positively charged anti-parallel four helix bundle where a fifth helix, helix α2, protrudes away from the helical core.

**Table 1:** Data collection and refinement statistics (molecular replacement).

Data collection and refinement	BBK32_(206–348)_
Data collection	
Space group	*P*6_5_
Cell dimensions	
*a*, *b*, *c*, Å	66.53, 66.53, 79.51
α, β, γ,°	90.00, 90.00, 120.00
Resolution, Å	33.3–1.72 (1.78–1.72)
*R*_pim_	0.019 (0.397)
*I/σI*	35.3 (1.9)
Completeness, %	99.8 (99.9)
Redundancy	22.1 (16.8)
Refinement	
Resolution, Å	33.3–1.72
No. reflections	21,115
*R*_work_/*R*_free_	20.5 / 23.6
No. non-hydrogen atoms	1,265
Protein	1,149
Water	116
*B*-factors	
Protein	41.98
Water	50.66
Rmsd	
Bond lengths, Å	0.007
Bond angles,°	0.96

**Fig 7:**
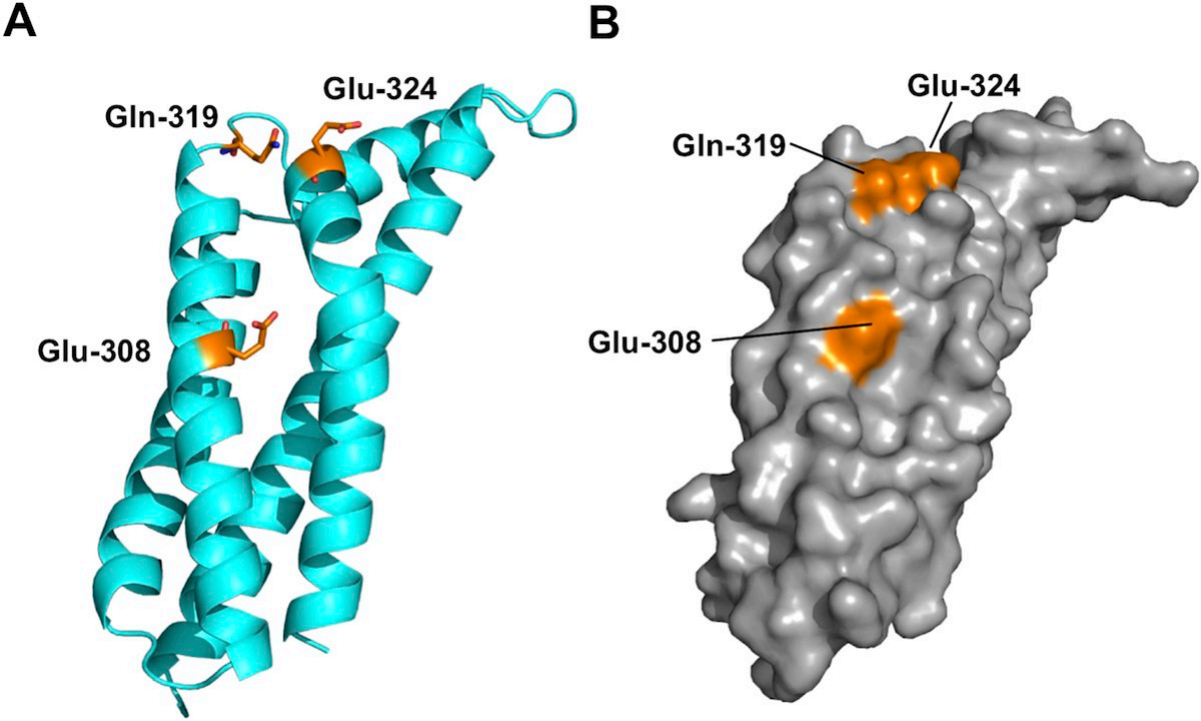
Residues in the BBK32 to BGD19 chimera are solvent exposed. A) A chimeric BBK32-C protein encoding three charged residues which are identical between BBK32 and BAD16, but different in BGD19-C, exhibits BDG19-like activity (see sequence alignment in Fig 1B and Fig 3). The structure of BBK32-C is oriented to highlight each of these residues (colored orange, stick representation). B) A molecular surface representation of BBK32-C in the same orientation as shown in panel A indicates all three residues altered in the BXK32-C chimera construct are surface exposed in the BBK32-C crystal structure.

The relative activities of the BXK32-C mutant suggest that one or more of the non-conservative amino acid substitutions between BBK32/BAD16 and BGD19 (i.e. E308, Q319, and E324) contribute to C1r inhibitory activity (**Fig 3**). In the BBK32-C structure E308 is a surface exposed residue located midway through α4, while Q319 and E324 are in the short loop connecting α4 and α5. Q319 and E324 are also surface exposed and together present a contiguous surface region (**Fig 7A and 7B**). Homology models of BAD16-C, BGD19-C, and BXK32-C were constructed using SWISS-MODEL and templated on the BBK32-C crystal structure (**S5 Fig**, PBD: 6N1L). These models predict that residues at the 308, 319, and 324 positions (BBK32 numbering) [40,41] are also surface exposed in each BBK32 orthologue protein (**S5 Fig**). As each of these residues is exposed to solvent, they are potentially positioned to interact with C1r, supporting the functional data implicating their involvement in BBK32-mediated complement inhibitory activity (**Fig 3**).

### The C1r-SP domain is required for high affinity interaction with BBK32-C

**Fig 8:**
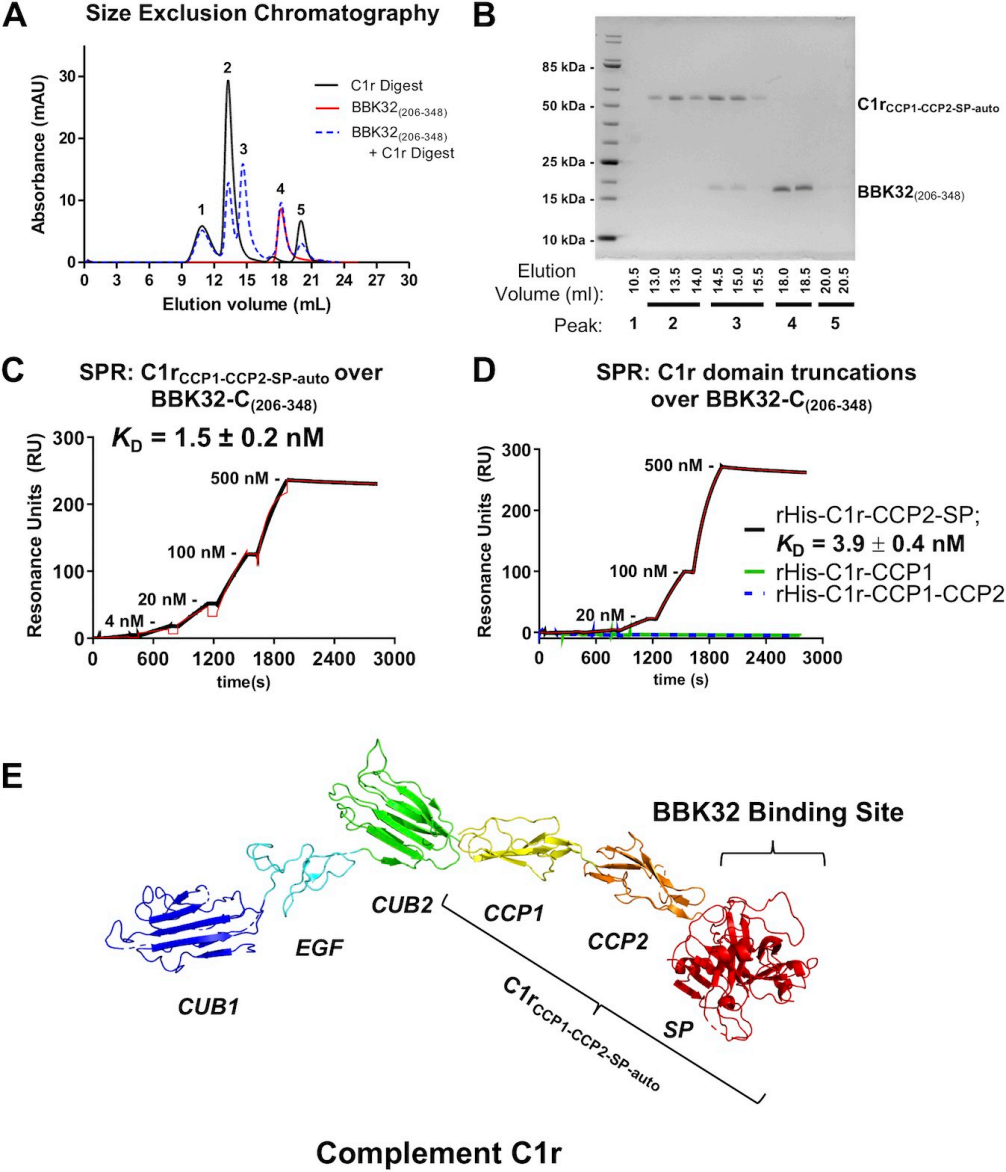
BBK32 binds to the C-terminal region of C1r and requires the SP domain for high affinity interaction. A-B) Intrinsic proteolysis of C1r reaches completion upon overnight incubation at 37°C resulting in the release of a fragment corresponding to the C-terminal domains CCP1-CCP2-SP. The auto-catalyzed digestion reaction of C1r was injected onto a size exclusion column. The C1r_CCP1-CCP2-SP-auto_ proteolytic fragment elutes in peak 2. When BBK32_(206–348)_ is added to the C1r digestion reaction at 2-fold molar excess (relative to full-length C1r) a new peak appeared, peak 3, which contains both BBK32_(206–348)_ and the C1r_CCP1-CCP2-SP-auto_ proteolytic fragment, as judged by mass spectrometry analysis. C) To confirm that BBK32 recognizes the C-terminal C1r CCP-1-CCP2-SP domains, SPR binding studies were performed. Purified C1r_CCP1-CCP2-SP-auto_ exhibited high affinity interaction for BBK32_(206–348)_ (*K*_D_ = 1.5 nM) D) Recombinant refolded His-C1r-CCP2-SP retains high affinity interaction (*K*_D_ = 3.9 nM), whereas recombinant His-CCP1 or His-CCP1-CCP2 alone fail to interact with BBK32_(206–348)._ E) A model of full-length C1r is shown which is built from the available crystal structures of C1r domain truncations (PDB’s: 4LOT, 6F39, and 1GPZ). The location of the C1r_CCP1-CCP2-SP-auto_ proteolytic fragment is indicated. Together these data indicate that BBK32 targets the C-terminal region of the C1r protease and requires the SP domain for high-affinity interaction.

The crystal structure of BBK32_(206–348)_ and identification of surface residues which affect C1r inhibition presented above provides insight into the structural determinants for BBK32-mediated C1r recognition. However, the C1r domains involved in mediating BBK32/C1r complex formation are unknown. C1r is a 92 kDa chymotrypsin-like serine protease with a modular architecture consisting of two complement C1r/C1s, Uegf, Bmp1(CUB) domains, an epidermal growth factor (EGF) domain, two complement control protein (CCP) domains, and a serine protease (SP) domain arranged in sequential fashion (CUB1-EGF-CUB2-CCP1-CCP2-SP) (**Fig 8**). We noted that purified full-length C1r has been reported to undergo a series of autoproteolytic cleavages when incubated for prolonged periods [42,43]. Following overnight incubation of purified C1r at 37°C, we injected the autolytic C1r digestion reaction onto a size exclusion chromatography column (**Fig 8A**, black line). The chromatogram displayed three well-resolved peaks (labeled 1, 2, and 5). Next, purified BBK32_(206–348)_ was injected alone resulting in a single peak (labeled 4) (**Fig 8A**, red line). Finally, a 2-fold molar excess of BBK32_(206–348)_ was mixed with the autoproteolytic C1r digestion reaction and injected onto the column (**Fig 8A,** blue dashed line). While peaks 1, 2, 4, and 5 remain, a new peak also appears (labeled peak 3). Analysis of each peak in the BBK32_(206–348)_/C1r digestion injection was performed using non-reducing SDS-PAGE (**Fig 8B**). A single band which migrates on the gel at an apparent molecular mass of 55 kDa was found in peak 2. This same 55 kDa band co-eluted with BBK32_(206–348)_ in peak 3. Mass spectrometry analysis identified the 55 kDa band observed in peaks 2 and 3 to be identical to one another and to correspond to residues Leu-300 to Asp-705 of C1r. This region maps to the C-terminal portion of C1r and includes the C-terminal six residues of the CUB2 domain and the entirety of the CCP1-CCP2-SP domains (hereafter referred to as C1r_CCP1-CCP2-SP-auto_). The C1r_CCP1-CCP2-SP-auto_ fragment identified here closely matches the previously reported autolytic C1r fragment known as γ-B [42,43]. As expected, the lower band observed on the gel which migrates at ~17 kDa in peak 3, was confirmed as BBK32_(206–348)_ by mass spectrometry analysis.

Co-migration of BBK32_(206–348)_ with the C1r_CCP1-CCP2-SP-auto_ proteolytic fragment suggested that BBK32 binds to the C-terminal region of C1r. To confirm this, we purified C1r_CCP1-CCP2-SP-auto_ and used SPR to assay its affinity for BBK32. Indeed, this autolytic C1r fragment displays similar affinity for BBK32 (**Fig 8C,** *K*_D_ = 1.5 nM) to that previously measured for full-length C1r (**Fig 2D**). To further refine the mapping of the BBK32 binding site on C1r we produced recombinant C1r domain truncations corresponding to the C-terminus of C1r. While C1r-CCP1 and C1r-CCP1-CCP2 failed to interact with BBK32 in SPR binding experiments, a construct containing only the CCP2-SP domains bound with similar affinity to BBK32 (**Fig 8D**, *K*_D_ = 3.9 nM) as was found for C1, C1r, and C1r _CCP1-CCP2-SP-auto_. Efforts to produce a recombinant protein corresponding to the C1r SP domain only were unsuccessful. However, collectively the data presented above strongly suggests that the SP domain of C1r is required for high affinity interaction with BBK32. A model of full-length C1r was constructed from the available C1r domain truncation mutants crystal structures [44–46], and the proposed BBK32 binding site is shown (**Fig 8E**).

## Discussion

Human complement is an evolutionarily ancient arm of the innate immune system that was first described nearly 120 years ago. Historically, complement has been regarded as a ‘first-line-of-defense’ against invading pathogens. Indeed, if a pathogen is unable to evade detection by one of the three complement pathways, initiating serine proteases begin converting zymogen complement proteins into activated fragments resulting in distinct but synergistic host defense mechanisms that include: i) opsonization (C1q, C3b, C4b); ii) phagocyte recruitment (C3a, C5a); iii) priming of the adaptive immune system (C1q, C3b, C4b, C3a, C5a); and iv) lysis (membrane attack complex). In this context it is no surprise that microorganisms that encounter blood, and other complement containing fluids, have evolved mechanisms to evade complement recognition and activation. Lyme disease spirochetes of the *Borrelia burgdorferi sensu lato* complex are among a group of human pathogens that have evolved several mechanistically distinct extracellular complement inhibitor proteins [5,7,22]. For example, *B*. *burgdorferi sensu lato* species employ a well-known pathogenic anti-complement strategy via expression of proteins which recruit the endogenous host regulator of the alternative pathway of complement, factor H, to the bacterial surface [5–12,47,48]. Lyme-associated *Borrelia* also produce a distinct set of surface proteins capable of binding host plasminogen and specifically degrading complement components [17–20], proteins that prevent complement activation at the level of C4 cleavage [21,49], and those that interfere with the formation of the membrane attack complex [14–16].

While complement has traditionally been viewed as a sentinel against microbial intruders, it is no longer considered an isolated innate immune response. Complement is integral to homeostatic maintenance and has direct roles in the regulation of both T cell and B cell immunity [50–52]. Interestingly, it has been hypothesized that the dominant function of some microbial complement inhibitors may be to interfere with complement-dependent shaping of adaptive immune responses, rather than protection from complement-mediated lysis [53]. In our recent infectivity studies, we noted that when mice are genetically deficient in the classical complement pathway pattern recognition molecule C1q they exhibit altered T cell and B cell responses to *B*. *burgdorferi* infection compared to wild type mice [54]. This is of potential relevance to BBK32-mediated classical pathway evasion as it has been shown that abrogated deposition of C4 on follicular dendritic cells underlies diminished antigen presentation and alters the kinetics of germinal center formation during Lyme borreliosis [55,56]. Given that C4 is one of two native substrates for C1, and that BBK32 directly inhibits C1 activation, it is possible that BBK32 may contribute to the impairment of germinal center formation and therefore the quality of antibody response to *B*. *burgdorferi* infections. Future studies will be important to elucidate the *in vivo* role of BBK32-mediated classical pathway complement inhibition on the subversion of T-dependent B cell responses by Lyme disease-causing spirochetes.

In regards to human serum susceptibility, *B*. *burgdorferi* and *B*. *afzelii* have been classified as resistant whereas *B*. *garinii* strains have often been classified as sensitive [36]. Differences in the susceptibility of *Borrelia burgdorferi sensu lato* species to complement may reflect an *in vivo* selection process that contributes to the pathogens ability to colonize different reservoirs [57–59]. For instance, a small rodent, *Peromyscus leucopus*, is the natural reservoir for *B*. *burgdorferi* in the Midwest and northeastern United States [60] whereas in Europe rodents and migratory birds are the principal reservoirs for *B*. *afzelii* and *B*. *garinii*, respectively [61]. For this study, we selected human serum-resistant strains of *B*. *burgdorferi* (strain B31) and *B*. *afzelii* (strain PGau), as well as a human serum-sensitive strain of *B*. *garinii* (strain IP90), to investigate the relative complement inhibitory activities of BBK32 orthologues. Quantitative affinity measurements using SPR with purified proteins indicated that recombinant BGD19-C binds C1r with similar affinity to that of BBK32-C and BAD16-C (**Fig 2**). Surprisingly, BGD19-C showed slightly weaker inhibition in complement assays involving artificial surfaces (**Fig 3A and 3B**), and conferred significantly reduced protection from complement-mediated lysis to the naïve membranes of sheep red blood cells (**Fig 3C**). These results suggest that binding of recombinant C-terminal BBK32 orthologues is necessary but not sufficient for potent C1r inhibitory activity. However, we note that when full-length BGD19 was expressed on the surface of a surrogate *B*. *burgdorferi* strain, it bound C1r weakly in qualitative binding assays relative to either full-length BBK32 or BAD16. It is unclear why C1r-binding observed in these assays differs from the similar affinities measured with recombinant proteins using SPR. Nonetheless, consistent with the weaker complement inhibitory properties of recombinant BGD19-C, surface expressed full-length BGD19 failed to protect *B*. *burgdorferi* B314 from complement-mediated killing (**Figs 4 and 5**). Collectively, our results show that *B*. *garinii* BGD19 has significantly reduced capacity to inhibit *in vitro* classical pathway complement activation compared to *B*. *burgdorferi* BBK32 or *B*. *afzelii* BAD16.

Our data support the notion that the relative increased susceptibility of *B*. *garinii* to human serum killing is related to the reduced activity of borrelial complement evasion proteins, as has been previously proposed for *B*. *garinii* factor H-binding proteins [37]. However, the *in vitro* serum-sensitivity classification scheme is recognized as being dependent on reagents, experimental conditions, and importantly the strains being studied [23]. Like *B*. *burgdorferi* and *B*. *afzelii*, *B*. *garinii* also causes human infections, and thus, some *B*. *garinii* strains can overcome complement-mediated clearance *in vivo*. *Borrelia burgdorferi sensu lato* spirochetes, including *B*. *garinii*, likely have multiple layers of functional redundancy that make up its complement evasion repertoire *in vivo*. Thus, while the reduction in activity of a single complement inhibitor like the BBK32 orthologue BGD19 is expected to contribute to the relative ability of *B*. *garinii* to survive complement-mediated attack *in vivo*, it must be considered in the context of a functionally redundant borrelial complement evasion system. Ultimately it will be the collective activities of these inhibitors, rather than dominance by a single complement evasion molecule, that would be expected to drive *in vivo* susceptibility of *Borrelia burgdorferi sensu lato* spirochetes to complement.

The crystal structure of *B*. *burgdorferi* BBK32-C presented here has provided the first insight into the structural determinants required for high affinity C1r interaction and inhibition by borrelial BBK32-like proteins. We probed the BBK32 molecular surface using a chimeric BBK32-C construct encoding three non-conserved surface residues originating from BGD19 (i.e. BXK32-C) and found that these substitutions alone shift the inhibitory activity of BBK32 towards that of BGD19. BBK32-like sequences are unique to the *Borrelia* genus and include three families of proteins found in relapsing fever-associated spirochetes termed FbpA, FbpB, and FbpC [62]. The relative sequence conservation of Fbp proteins to BBK32 is much lower than that of *B*. *burgdorferi sensu lato* orthologues and ranges between 25% and 60% identity at the amino acid level. Our data indicate that subtle changes in amino acid sequences can result in significant differences in the ability of *B*. *burgdorferi sensu lato* BBK32 orthologues to block human complement and thus it will be important to determine if BBK32-like classical pathway complement inhibition is restricted to Lyme disease spirochetes or is common to all pathogenic *Borrelia*.

*B*. *burgdorferi* BBK32, as well as its orthologues, have unique and apparently disparate functions within the vertebrate host. The disordered N-terminal half acts as an adhesin by binding to glycosoaminoglycans and the extracellular matrix protein fibronectin, while the ordered carboxy terminal half acts as a C1r-binding complement inhibitor (**Fig 1A**). While discrete BBK32-binding sites have been identified for each of these host ligands, it remains unknown if BBK32 interacts simultaneously with C1r and fibronectin or GAGs. This may be of importance as the formation of functionally synergistic ternary complexes involving virulence factors from other pathogens, such as *Staphylococcus aureus* extracellular fibrinogen-binding protein (Efb) in complex with complement C3 and fibrinogen, have been described [63]. Furthermore, linkage of an intrinsically disordered host-interaction domain with an ordered host interaction domain, like that observed in BBK32, is seen in Efb and several Gram positive MSCRAMMs [63–65]. Whether BBK32-like, covalently linked, intrinsically disordered/ordered structural domains with multifunctional host interaction properties is common in *Borrelia*–or even in other human pathogens–has yet to be fully evaluated.

Among the multi-pronged borrelial complement evasion arsenal [23], BBK32 is unique in its ability to specifically target the classical pathway of complement [22]. In fact, there are relatively few examples of pathogenic strategies which specifically target the classical pathway and BBK32 is the only known inhibitor which directly blocks the initiator protease C1r [66]. By localizing the BBK32 interaction site to the catalytically active serine protease domain on C1r and solving the high-resolution structure of BBK32-C in an unbound form, this study has greatly improved our knowledge of the molecular basis for BBK32-mediated C1r inhibition. Continued work in this area is needed to further refine the BBK32/C1r molecular interface and to pinpoint key residues that drive complex formation, knowledge of which will greatly improve our ability to harness the therapeutic potential of the potent and highly specific anti-complement activities of BBK32 proteins for use in complement-related diseases.

## Materials and methods

### Bacterial strains and plasmid constructs

*B*. *burgdorferi* B31 strains ML23 and B314, as well as *B*. *afzelii* strain PGau and *B*. *garinii* strain IP90, were grown in BSK-II media supplemented with 6% normal rabbit serum (Pel-Freez Biologicals, Rogers, AR) under microaerobic conditions at 32˚C, 1% CO_2_ atmosphere, pH 7.6. Strain B314 is a serum-sensitive, non-infectious strain B31 derivative that lacks most linear plasmids [67,68]. All *B*. *burgdorferi* cells were enumerated by dark field microscopy.

Heterologous *bbk32* genes from *B*. *afzelii* strain PGau and *B*. *garinii* strain IP90, designated as *bad16* and *bgd19*, respectively, were cloned into the shuttle vector pBBE22*luc*. To carry this out, oligonucleotide primers were designed based on the sixteenth open reading frame of lp17 from *B*. *afzelii* strain PKo (Genbank accession number CP002942.1; region 12854–13912 of lp17 from *B*. *afzelii* PKo) and the nineteenth open reading frame of lp17 from the *B*. *garinii* strain PBr (Genbank accession number CP001309.1; region 12206–11160 of lp17 from PBr). The letter “D” or “d” used to denote the orthologous protein or gene, respectively, is due to their presence on the lp17 episome, which is referred to as the “D” plasmid in *B*. *burgdorferi* strain B31 [69]. Note that the corresponding proteins from both *B*. *afzelii* strains are 100% identical whereas the *B*. *garinii* proteins share 96% identity. Oligonucleotide primers were synthesized by Eurofins, Inc. (Lousville, KY) and their corresponding sequences are shown in **Table 2**. Oligonucleotide primers with sequences that overlapped with the borrelial gene and the vector pBBE22*luc* were used for PCR amplification using genomic DNA from *B*. *afzelii* strain PGau and *B*. *garinii* strain IP90 as template. The amplified fragments contained 395 and 491 bp of upstream sequences and 185 and 177 bp downstream from the translational start site and stop codon corresponding to the 1059 bp *bad16* and 1065 bp *bgd19* genes, respectively. The resulting PCR products were 1639 bp and 1733 bp for *bad16* and *bgd19*, respectively. The plasmid pBBE22*luc* was digested with *Bam*HI HF and *Sal*I HF (New England Biolabs, Ipswich, MA) and assembled separately with each of the aforementioned PCR fragments using the manufacturer’s instructions for NEBuilder (New England Biolabs). The resulting constructs were transformed into *Escherichia coli* DH5α cells (F^–^ϕ80*lac*ZΔM15 Δ(*lac*ZYA-*arg*F)U169 *rec*A1 *end*A1 *hsd*R17(r_K_^–^, m_K_^+^) *pho*A *sup*E44 λ^–^ *thi*-1 *gyr*A96 *rel*A1) and transformants selected on LB agar plates containing kanamycin at 50 μg/ml (Sigma-Aldrich; note that all chemicals and reagents mentioned herein were purchased from Sigma-Aldrich unless indicated otherwise). The resulting constructs, which contained *bad16* and *bgd19* expressed under the control of their native promoters, were confirmed by sequencing and designated pBAD16 and pBGD19, respectively.

**Table 2:** Oligonucleotides used in this study.

Oligo- nucleotide	Sequence (5’ to 3’)	Description	Refer-ence
pBBE22-bad16usF	GGATAGCATAGAGGTACCCGGGGATCCCAAACCTAAATATGGTCTTAAAGTAAAGATAG	Oligonucleotide pair used to amplify 1639 bp containing *bad16* and the upstream/down region from *B*. *afzelii* PGau with a 27 bp 5' and a 22 bp 3' flanking region homologous to pBBE22luc vector, including the *Bam*HI site *Sal*I sites.	This study
bad16ds- pBBE22R	GCTTGCATGCCTGCAGGTCGACCATATTCTGATATATCCTGTAAACAGTGTT		
pBBE22- bgd19usF	GGATAGCATAGAGGTACCCGGGGATCCTTAGCAGCAACTGAAAAATTAGACAAAGC	Oligonucleotide pair used to amplify 1733 bp containing *bgd19* and the upstream/down region from *B*. *garinii* IP90 with a 27 bp 5' and a 22 bp 3' flanking region homologous to pBBE22luc vector, including the *Bam*HI site *Sa*lI sites.	This study
bgd19ds- pBBE22R	GCTTGCATGCCTGCAGGTCGACAATTCTGATATAGCTTAAACAATATTTTTGAC		
*pnc*Af	TATTGGAATTAATAGGCGGTGATG	Oligonucleotide pair used to confirm pBAD16, pBGD19, and pCD100 constructs	This study
*luc*f	GAGGGGTTGTATTTGTTGACG		
qRT- bad16F	TGGTGAAAGTGGTGAATTGAAGG	Oligonucleotide pair used in the qRT-PCR to check for *bad16* expression in B314/pBAD16.	This study
qRT- bad16R	AGAATTTGAGCCTGAAATAGCTTG		
qRT-bgd19F	TTCCCTTAGCGGTGAAAGTGGTG	Oligonucleotide pair used in the qRT-PCR to check for *bgd19* expression in B314/pBGD19.	This study
qRT-bgd19R	CTTGATCCTGAAATGCCTTGTAGG		
flaBf	CAGCTAATGTTGCAAATCTTTTCTCT	Oligonucleotide pair used in the qRT-PCR to check for *flaB* expression in all the B314 background strains.	Hyde et al., 2007
flaBr	TTCCTGTTGAACACCCTCTTGA		
BBK32f	GAATATAAAGGGATGACTCAAGGAAGTT	Oligonucleotide pair used in the qRT-PCR to check for *bbk32* expression in B314/pCD100.	Hyde et al., 2007
BBK32r	TTTGGCCTTAAATCAGAATCTATAGTAAGA		

Transformation of strain B314 with pBAD16 and pBGD19 was done as previously described [70]. Transformants were selected for resistance to kanamycin and screened by PCR to confirm the presence of pBBE22*luc* vector. The cloning was confirmed with PCR and sequencing with primers *pnc*Af and *luc*f (**Table 2)**.

### Proteins

Recombinant BBK32-C (residues 206–354, *B*. *burgdorferi* strain B31), BBK32-C_(206–348)_ (residues 206–348, *B*. *burgdorferi* strain B31), BGD19-C (residues 206–354, *B*. *garinii* strain IP90), BAD16-C (residues 204–352, *B*. *afzelii* strain PGau), and BXK32-C (i.e. BBK32-C E308K-Q319K-E324Q) were sub-cloned into the pT7HMT vector [71] and purified to homogeneity using protocols previously described for BBK32-C [22]. For BBK32-C_(206–348),_ a DNA fragment encoding BBK32 residues 206 to 348 was generated by PCR from the existing pT7HMT-BBK32-C plasmid [22] using oligonucleotide primers that appended *Bam*HI and *Not*I sites at the 5’ and 3’ends, respectively_._ For BGD19-C, BAD16-C, and BXK32-C an *Escherichia coli* codon-optimized DNA sequence flanked with a 5’ *Bam*HI site, a 3’ *Not*I site, and a stop codon were synthesized commercially using IDT Technologies gBlock Gene Fragment service. Recombinant C1r domain truncations corresponding to the CCP1, CCP1-CCP2, or CCP2-SP domains (as defined by UNIPROT ID: P00736) were produced as follows. Synthetic *E*. *coli* codon-optimized DNA (synthesized by IDT Technologies gBlock), corresponding to the sequence of human C1r domains CCP1-CCP2-SP (amino acid residues 307–705), was used as a PCR template along with oligonucleotide primers that appended *Bam*HI and *Not*I at the 5’ and 3’ends, respectively, to produce DNA fragments corresponding to CCP1 (residues 309–373), CCP1-CCP2 (residues 309–449), and CCP2-SP (residues 376–705). All C1r domain truncations were purified under denaturing conditions using previously published protocols [71] with the following modifications. CCP1 and CCP1-CCP2 were refolded by overnight dialysis at room temperature into 100 mM Tris (pH 8.6), 20 mM glycine, 1 mM ethylenediaminetetraacetic acid (EDTA), 1mM L-cysteine, and 2.5 M Urea. A second overnight dialysis was then performed into 20 mM Tris (pH 8.0), 10 mM Imidazole, 500 mM NaCl and further purified using nickel affinity and gel filtration chromatography using a HiLoad Superdex 75 PG column (GE Healthcare) CCP2-SP was refolded according to previously published protocols [72]. Briefly, 5 ml’s of the CCP2-SP denaturing nickel affinity eluent was rapidly diluted into 50 ml’s of a buffer containing 50 mM Tris (pH 8.3), 3 mM reduced glutathione/1mM oxidized glutathione, 5 mM EDTA, and 500 mM L-arginine and allowed to incubate overnight at room temperature. This step was followed by overnight dialysis into 50 mM Tris (pH 7.4), 145 mM NaCl. Following refolding, CCP2-SP was further purified by gel filtration chromatography using a HiLoad Superdex 200 PG column (GE Healthcare). Purified C1 complex and full-length C1r enzyme were obtained from Complement Technology (Tyler, TX). Biotinylation of C1 and C1r was done as previously described [22].

### Surface plasmon resonance

All SPR experiments were conducted on a Biacore T200 instrument at 25°C and unless otherwise noted using a flowrate of 30 μl min^-1^ and a running buffer of HBS-T (20 mM HEPES (pH 7.3), 140 mM NaCl, 0.005% Tween-20). Proteins were immobilized using standard amine coupling chemistry on CMD200M biosensor chips (Xantec) as described previously [22]. The following immobilization densities were used for the corresponding injection series: C1 analyte over BBK32-C (680 RU), BGD16-C (850 RU), BAD16-C (720 RU); C1r analyte over BBK32-C (1800 RU), BGD19-C (4060 RU), BAD16-C (3200 RU); C1r_CCP1-CCP2-SP-auto_ analyte over BBK32_(206–348)_ (780 RU, 1760 RU, 1600 RU). The C1 and C1r injection series were performed in HBS-T buffer supplemented with 5 mM CaCl_2_. C1 injections consisted of a twelve point, two-fold dilution series ranging from 0 to 150 nM C1 for 2 min association and 3 min dissociation. C1r was injected using a single-cycle kinetic format [73] using a five point, five-fold dilution series ranging from 1.6 to 1000 nM. Regeneration to stable baseline was achieved by injecting HBS-T supplemented with 10 mM EGTA for 1 min followed by three 30 s injections of a solution containing 0.1 M glycine (pH 2.2), 2.5M NaCl. C1r_CCP1-CCP2-SP-auto_, C1r_CCP1_, C1r_CCP2,_ and C1r_CCP1-CCP2-SP_ injections were identical to that of C1r using a concentration range of 0.8 to 500 nM. Kinetic analysis was performed for each set of sensorgrams injections using T200 Evaluation Software (GE Healthcare) using a 1:1 (Langmuir) binding model and a dissociation constant (*K*_D_) was calculated from the resulting fits. All injection series were performed in triplicate and the mean value is reported for each *K*_D_ ± standard deviation.

### Complement inhibition assays

The ability of recombinant BBK32 orthologue proteins to inhibit the activation of human complement was assessed using two assay formats. First, an ELISA-based assay was used that relies on the activation of the classical pathway via surface-immobilized IgM (Athens Research and Technology) and subsequent detection of the complement deposition products derived from normal human serum (Innovative Research), specifically C3b or MAC through use of monoclonal antibodies (both purchased from Santa Cruz Biotechnology) [74]. Each borrelial protein was evaluated using a duplicate 12-point, two-fold dilution series ranging from 2 to 2000 nM. A second assay was used which monitors the hemolytic activity of human complement activation via the classical pathway in the presence of recombinant BBK32-C or the BBK32 orthologues BAD16-C, BGD19-C, or BXK32-C by using sensitized sheep erythrocytes (Complement Tech, Tyler, TX). In each case these assays were performed in an identical manner to those described previously in detail for the evaluation of BBK32-C [22].

### Proteinase K accessibility assay

*B*. *burgdorferi* strains B314/pBAD16 and B314/pBGD19 were grown in complete BSK-II media, harvested by centrifugation at 5,800 x *g*, and washed twice with PBS. The cell pellet was re-suspended in 0.5 ml of either PBS alone or with PBS with proteinase K (Invitrogen) to a final concentration of 200 μg ml^**-**1^. All samples were incubated at 20°C for 40 min. Reactions were terminated by the addition of phenylmethylsulfonyl fluoride (PMSF) to a final concentration of 1 mM. Cells were again pelleted by centrifugation (9,000 x *g* for 10 min at 4°C), washed twice with PBS containing 1 mM PMSF, re-suspended in Laemmli sample buffer, and resolved on SDS-PAGE. The separated proteins were transferred to a PVDF membrane (Thermo Fisher Scientific) and probed as described below with anti-BAD16, anti-P66, and anti-FlaB antibodies, respectively.

### *B*. *burgdorferi* whole cell adherence assays

*B*. *burgdorferi* adherence assay was done as previously described with slight modifications [22,75]. Briefly, poly-D-lysine pre-coated coverslips (Corning Biocoat) were coated with 1 μg human C1r (Complement Tech) or BSA, respectively, and incubated at 4°C overnight. The coverslips were washed thoroughly in PBS to remove excess unbound proteins. The coverslips were then blocked with 3% BSA at room temperature for 1 hr. *B*. *burgdorferi* strains B314/pBBE22*luc* (vector only control), B314/pCD100 (expresses *bbk32*), B314/pBAD16 (expresses *bad16*), and B314/pBGD19 (expresses *bgd19*) were grown to mid-logarithmic phase at 32°C, 1% CO_2_, pH 7.6. All *B*. *burgdorferi* strains were subsequently diluted to 10^7^ organisms/ml in BSK-II medium without serum. The resulting *B*. *burgdorferi* samples, in 0.1 ml volumes, were applied onto the coverslips and incubated for 2 hr at 32°C. Unbound bacteria were removed from the coverslips by gentle washing with PBS; this wash step was repeated 7 times. The coverslips were applied to a glass slide and the binding of spirochetes was scored by dark field microscopy.

### Serum complement sensitivity assay

Complement sensitivity assays were performed as previously described [22]. Briefly, *B*. *burgdorferi strains* were grown to exponential phase at 32°C, 1% CO_2_, pH 7.6, and 80 μl of a 10^6^ cell suspension in BSK-II medium was added to 20 μl of normal human serum (NHS; Complement Technologies) to give a final volume of 100 μl (i.e., 20% NHS). The samples were placed in microtiter plates and the suspensions were sealed and incubated at 32°C for 2 h. Heat-inactivated normal human serum (hiNHS) was used as a control. After incubation, *B*. *burgdorferi* cells were scored by dark field microscopy and the percentage of viable *B*. *burgdorferi* cells were calculated from randomly chosen fields and based on immobilization, loss of cell envelope integrity, and/or overt lysis.

### Crystallization, structure determination, refinement, and analysis

BBK32_(206–348)_ was concentrated to 5.1 mg ml^-1^ in a buffer of 10 mM HEPES (pH 7.3), 50 mM NaCl. Crystals of BBK32_(206–348)_ were obtained by vapor diffusion of sitting drops at 20°C. Drops were setup by mixing 1 μl of protein solution with 1 μl of precipitant solution. Two crystallization conditions were identified. The first condition contained 0.1 M MES (pH 6.5), 0.2M ammonium sulfate, and 30% PEG-MME 5,000. Small plate clusters reproducibly appeared in this condition between 2 and 5 d with rounds of microseeding producing large plates which could be harvested and cryoprotected with supplementation of 5% glycerol to the precipitant solution. Crystals in this condition grew in the space group *P*2_1_ with four BBK32_(206–348)_ molecules in the asymmetric unit, diffracting to 2.5 Å resolution. A second condition was identified containing 15% PEG 3,350 and 0.1M succinic acid (pH 7.0). These crystals appeared only after prolonged incubation (i.e., > 6 months). Cryoprotection was achieved by supplementing the precipitant solution with 20% glycerol. These crystals grew in space group *P*6_5_ with a single copy of BBK32_(206–348)_ in the asymmetric unit, diffracting to 1.7Å. Monochromatic X-ray diffraction data were collected at 0.973-Å wavelength using beamline 22-ID of the Advanced Photon Source (Argonne National Laboratory). Diffraction data were integrated, scaled, and reduced using the HKL2000 software suite [76]. Of all deposited structures in the RCSB database, none share > 25% sequence identity to BBK32-C. Exhaustive attempts at various molecular replacement strategies utilizing the *P*2_1_ dataset failed. However, a single solution was found using the *P*6_5_ dataset by the MRage program [77] implemented via the PHENIX crystallography software suite [78–80]. MRage was configured to use a homology search based on the top three hits for BBK32 obtained from the HHPRED server implemented via the MPI Bioinformatics Toolkit [81]. The top scoring solution was a homology model of 51 residues (BBK32 residues 267–317) based on a partial structure of PDBID: 5J0K [82]. Despite a relatively low scoring solution (LLG = 43.8, TFZ = 6.6), initial phases obtained from this search, yielded an initial PHENIX.AUTOBUILD model which refined to 24%/27% (R_work_/R_free_). Subsequent manual building was performed using COOT [83] and iterative cycles of refinement using PHENIX.REFINE produced a final refined model of 20.5%/23.6% (R_work_/R_free_). Residues 206–208 are missing from the final model due to poor electron density and thus the completed model contains BBK32 residues 209–348. Using the final refined model obtained from this crystal as a molecular replacement search model readily provided a solution for the *P*2_1_ crystal and a final refined model of 20.8% / 26.9% (R_work_/R_free_). Refined coordinates and structure factors for the *P*6_5_ crystal form have been deposited in the Protein Data Bank, Research Collaboratory for Structural Bioinformatics, Rutgers University (www.rcsb.org/) under PDB ID code 6N1L. A description of crystal cell constants, diffraction data quality, and properties of the final model for BBK32_(206–348)_ can be found in Table **1**. Representations of protein structures and electron density maps were generated by PyMol (www.pymol.org/). Homology models of BGD19-C, BAD16-C, and BXK32-C presented in S5 Fig were created using SWISS-MODEL in user-template mode where the crystal structure of BBK32-C (PDB:6N1L) served as a template.

### C1r autolytic digestion and BBK32-binding site analysis

A total of 400 μl of purified C1r (Complement Tech) at 1.0 mg ml^-1^ was diluted into an equal volume of 50 mM Tris (pH 8.0), 0.5 mM CaCl_2_ and allowed to incubate overnight at 37°C. 150 μl of this reaction was mixed with either 100 μl of buffer or 100 μl of BBK32_(206–348)_ at 2 fold molar excess relative to full-length C1r. A third sample was prepared with only BBK32_(206–348)_. Each 250 μl sample was then injected onto a SuperDex 200 Increase 10/300 GL small scale size-exclusion column (GE Healthcare) previously equilibrated in 10 mM HEPES (pH 7.3), 140 mM NaCl at a flowrate of 0.5 ml min^-1^. Peaks were evaluated by SDS-PAGE under non-reducing conditions. The bands in ‘Peak 3’ (elution volume 14.5 to 15.5) were identified by mass spectrometry.

Gel bands representing the autolytically formed 55kDa form of C1r, and BBK32_(206–348)_ were excised from the SDS-PAGE gel, reduced and alkylated, and digested with trypsin overnight by standard methods. Extracted peptides from each digest were subjected to LC-tandem MS analysis for verification and determining the coverage of C1r in the 55kDa gel band. The digests were resolved by reversed phase nanoLC in a C18 column (50μm x 12cm, packed with Phenomenex Jupiter, 10μm), in a 1% to 35% acetonitrile, 100 minute gradient (buffer A, 0.1% Formic acid in water), eluting into a Q Exactive Plus MS system. Data were acquired in data dependent mode, MS scans at 35k, with 16 dependent MS2 scans per cycle at 17.5k resolution. The HRMS data files were searched using Mascot version 2.6 against a custom database consisting of the full length native human C1r sequence (Uniprot accession P00736), and a sequence for BBK32_(206–348)_ consistent with the predicted amino acid sequence of the cloned construct. Peptides considered were restricted to semi-Trypsin specificity, with tolerances of 10ppm (MS) and 0.01Da (MS2 fragment) allowed, with fixed Carbamidomethyl (Cys), and variable modifications Deamidation (Asn,Gln) and Oxidation (Met) included. Peptides identified at P > 0.05 threshold were manually inspected to verify the quality of the apparent sequence coverage.

### Far Western and conventional immunoblotting

In the Far Western assay, three biological repeats were performed for each *B*. *burgdorferi* strains, B314 (no vector), B314/pBBE22*luc* (vector only control), B314/pCD100 (produces BBK32), B314/pBAD16 (produces BAD16), and B314/pBGD19 (produces BGD19). The protein lysate of one repeat of each *B*. *burgdorferi* strain was resolved in the same SDS–PAGE gel and transferred to PVDF membranes as described [22,84]. PVDF membranes were blocked in 5% non-fat milk (Wal-Mart Stores, Inc.) in PBS, 0.2% Tween-20, washed with PBS, 0.2% Tween-20, and then incubated with biotinylated C1 or C1r (both from Complement Tech) at 1 μg/ml in PBS, 0.2% Tween-20. The blot was incubated with rocking overnight at 4°C. The membrane was then washed in PBS, 0.2% Tween-20 and subsequently incubated with infrared fluorescent dye (IRDye) 800CW Streptavidin (Li-Cor Biosciences; Lincoln, NE) diluted 1:1000 in 5% non-fat milk, 0.2% Tween-20 with 0.1% SDS for 1 hr. Subsequently, the membrane was washed extensively in PBS, 0.2% Tween-20 and scanned using the Li-Cor Odyssey Fc Imaging System.

Immunoblotting to detect the endoflagellar antigen FlaB was done for all samples using the same PVDF membrane used in C1 or C1r Far Western detection. A monoclonal to *B*. *burgdorferi* strain B31 FlaB (Affinity BioReagents) was diluted at 1:4,000 and incubated with the blot for 1 hr. After washing in PBS, 0.2% Tween-20, the blot was next incubated with a 1:10,000 dilution of Goat anti-mouse IgG with IRDye 680RD (Li-Cor Biosciences) as the secondary antibody. The membrane was washed extensively in PBS, 0.2% Tween-20 and scanned using the Li-Cor Odyssey Fc Imaging System.

The signals obtained from the Li-Cor unit were analyzed using the Image Studio-lite Ver 5.2.5 software. The bands were detected with manual adjustment to their shape relative to background. All BBK32 orthologue signals obtained were initially normalized to the FlaB signal from the same sample, then to the B314/pCD100 on the same blot, to compare across distinct Far Western blots quantitatively as indicated here for BAD16:

$$\mathrm{Signal}\;\mathrm{for}\;\mathrm{BAD}16=\frac{BAD16\;binding\;to\;C1/FlaB\;signal}{BBK32\;binding\;to\;C1/FlaB\;signal\;on\;the\;same\;membrane}$$

The resulting values were then used in statistical analyses described below.

Conventional immunoblots were also performed with rat polyclonal antibodies to BBK32-C, BAD16-C, and BGD19-C (each diluted 1:1000; kindly provided by Richard Marconi) produced in the B314 background strain. To detect membrane-bound immune complexes, Goat anti-Rat IgG with an IRDye 800CW conjugate (Li-Cor Biosciences) was used as a secondary antibody and diluted to 1:10,000. Detection of P66 was accomplished using rabbit anti-P66 serum (generously provided by Sven Bergström) diluted 1:1000 followed by detection on membrane immune complexes using a 1:10,000 dilution of Goat anti-rabbit conjugated with IRDye 800CW (Li-Cor Biosciences). The membranes were scanned using the Li-Cor Odyssey Fc Imaging System.

### Transcript quantification of *bbk32* and orthologues

Three independent cultures of each *B*. *burgdorferi* strain B314/pCD100 (expresses *bbk32*), B314/BGD19 (expresses *bgd19*), and B314/BAD16 (expresses *bad16*) were grown to the exponential growth phase (i.e., 5 x 10^7^ cells per ml), and total RNA was isolated from 5 x 10^8^ cells using Direct-zol RNA MiniPrep (Zymo Research, USA). The RNA samples were treated with the in-kit DNase I and TURBO DNA free kit (Invitrogen, USA) to eliminate contaminating DNA. RNA integrity was examined by gel electrophoresis.

Oligonucleotide primers for amplifying *flaB* and *bbk32* via quantitative RT-PCR (qRT-PCR) were adopted from prior studies [85] and primers for *bad16* and *bgd19* were designed in this study and shown in Table 2. Each primer pair was tested to confirm amplification of a single product of the expected size using genomic DNA from appropriate *B*. *burgdorferi* sensu lato strains as template. Reverse transcription reactions of three biological repeats of each strain were carried out with SuperScript II Reverse Transcriptase (Invitrogen, Carlsbad, CA). A control reaction with a mixture lacking reverse transcriptase was performed for each primer set to confirm that DNA was not present. Subsequently, the products from the reverse transcription reaction were subjected to quantitative real-time PCR using an Applied Biosystems StepOnePlus Real-Time PCR system. PowerUp SYBR Green Master Mix (Thermo Fisher Scientific) was used to perform quantitative PCR in triplicate (technical repeats). The constitutively expressed *flaB* gene of *B*. *burgdorferi* was used for normalization as previously described [85,86]. The expression levels of *bbk32* orthologues were first normalized to the *flaB* in the same sample, then the normalized values of *bad16* and *bgd19* were compared to the level of *bbk32* using the 2^-ΔΔCt^ method. The final fold differences were used in statistical analyses.

### Statistics

Statistical analysis was performed with GraphPad Prism version 7. For calculation of IC_50_ values in ELISA and hemolytic complement assays using recombinant proteins, non-linear regression was performed using a variable four-parameter fit where the top and bottom values were constrained to 100 and 0, respectively. Two-way ANOVA were used in C1r binding assay and serum sensitivity assay, and One-way ANOVA were used in Far Western and qRT-PCR analyses.

## Supporting information

S1 Fig BBK32-C mediated inhibition of the classical pathway of complement. A) A schematic depiction of classical pathway complement activation is shown. C1q, the pattern recognition subunit of the C1 complex, binds to the targeted surface. C1q binding autoactivates the initiator serine protease, C1r, which then proteolytically cleaves C1s. Activated C1s cleaves complement proteins C2 and C4 leading to the surface formation of C4b2a, the CP (Classical Pathway)/LP (Lectin Pathway) C3 convertase. C3 convertases then cleave C3 into C3a and C3b leading to CP/LP C5 convertase formation (C4b2a3b). Cleavage of C5 by C5 convertases releases the anaphylatoxin C5a and leads the formation of the membrane attack complex (C5b-9) on the surface of the target cell. The membrane attack complex is a lytic pore structure that can directly kill the targeted cell(s). For *Borrelia* species, BBK32, or active orthologues of BBK32, can block activation of C1r and inhibit the classical complement cascade. B) A model for BBK32-mediated inhibition of the classical pathway. C1 complex, consists of C1q, which is composed of six collagen-like structures connected to six globular head domains. C1q binds a C1r_2_C1s_2_ heterotetramer to form C1 complex. The depiction of the arrangement of subunits within C1 is based on the work of Ugurlar and colleagues [87]. BBK32-C, binds the exposed serine protease (SP) domain of C1r and inhibits the autoproteolytic activation of C1r as well as the C1r-mediated cleavage of proC1s. Inhibition at this step halts the classical pathway at the initial proteolytic step and prevents formation of the downstream activation products of the cascade, including the membrane attack complex.
(TIF)

S2 Fig Construction of *bbk32* orthologues into pBBE22*luc* and expression of these genes in *B burgdorferi* strain B314. A) Schematic showing how the *bbk32* orthologues *bad16* and *bgd19* from *B*. *afzelii* and *B*. *garinii*, respectively, were constructed using the pBBE22*luc* vector backbone. The resulting constructs were transformed into *B*. *burgdorferi* strain B314. B) PCR confirmation of *bgd19* from B314/pBGD19, *bbk32* from B314/pCD100, and *bad16* from B314/pBAD16. All constructs contained the *bad16*, *bbk32*, *bgd19* expressed from their native promoters. The Vector lane refers to the use of pBBE22*luc* as template for PCR with the oligonucleotide primers used to screen inserts. Values listed to the left indicate the size of markers in kilobases (kb). C) Quantitative RT-PCR shows that the expression of *bbk32* orthologues (e.g., *bad16* and *bgd19*) in strain B314 using their native promoters make transcripts equivalent or greater than *B*. *burgdorferi sensu stricto bbk32*. Expression of the *bbk32* orthologues was compared relative to the constitutively expressed *flaB* gene (internal control). The qRT-PCR was done in triplicate and the mean values obtained for *bbk32* was used as a comparator for the other orthologous genes (i.e., *bad16* and *bgd19*).
(TIF)

S3 Fig Cross-reactivity of BBK32 orthologues and evaluation of their surface exposure in *B burgdorferi* strain B314. A) Antisera to BBK32 orthologues is cross reactive against all *sensu lato* isolates tested. Antisera against BGD19 from *B*. *garinii*, BBK32 from *B*. *burgdorferi*, and BAD16 from *B*. *afzelii* were tested in immunoblots of protein lysates from *B*. *burgdorferi* strain B314 containing the vector pBBE22*luc* (B314/luc), as well as B314 strain expressing *B*. *garinii bgd19* (B314/pBGD19), *B*. *burgdorferi bbk32* (B314/pCD100), and *B*. *afzelii bad16* (B314/pBAD16). Individual membranes were then probed with rat polyclonal antisera against BGD19-C, BBK32-C, and BAD16-C as specified on the right. In all instances, the reagent used recognized its homologous target protein best but also showed significant reactivity to the other heterologous proteins. Markers in kDa are indicated on the left. B) The BBK32 orthologues encoded by *B*. *afzelii* and *B*. *garinii*, designated as BAD16 and BGD19, respectively, are surface exposed in the surrogate *B*. *burgdorferi* B314 strain. B314/pBAD16 and B314/pBGD19, encoding BAD16 and BGD19, respectively, were grown, washed, and then either resuspended with Proteinase K (ProtK; denoted with a “+”) or buffer alone (denoted with a “-“). Following processing, the resulting samples were subjected to SDS-PAGE and immunoblotted with antiserum directed against either BAD16-C, the outer membrane P66 protein, or the subsurface FlaB protein. Given the cross-reactivity of anti-BAD16 with all *B*. *burgdorferi* BBK32 orthologues (panel A), the fate of BGD19 could be assessed with the anti-BAD16-C reagent. Protein markers are indicted in the left (in kDa).
(TIF)

S4 Fig Validation of BBK32_(206–348)_ activity and electron density map quality. A-C) The construct used for crystallization, BBK32_(206–348)_, which lacks six C-terminal residues relative to BBK32-C (i.e. BBK32_206-354_), retains high affinity C1r interaction and complement inhibitory properties. D) 2*F*o-*F*c density contoured at 1.2 σ for the entire BBK32 polypeptide.
(TIF)

S5 Fig Three surface residues contribute to the reduced C1r-inhibitory profile of *B. garinii* BGD19. SWISS-MODEL was used to produce homology models of A) BAD16-C and B) BGD19-C that are based on the crystal structure of BBK32-C (PDB: 6N1L). Residues that are non-identical between BAD16-C and BBK32-C are shown in red on the protein surface (panel A), while residues that differ between BGD19-C and BBK32-C are shown in yellow (panel B). C) The homology models of BAD16-C and BGD19-C are structurally aligned. The coloring scheme shown in panels A/B is retained except overlapping residues are now colored in orange. Surfaces that remain yellow represent residues that are uniquely different in BGD19-C relative to BAD16-C. D) Three of these residues were selected for the BXK32-C chimera protein used in this study including residue positions 308, 319, and 324 (BBK32 numbering). A SWISS-MODEL homology model of the BXK32-C chimeric protein, also based on the BBK32-C crystal structure, predicts these three residues would remain solvent exposed. Global Model Quality Estimation (GMQE) is used by SWISS-MODEL to provide an estimate of model accuracy. Values range between 0 and 1, with higher numbers indicating higher model reliability and are as follows: BAD16-C (GMQE = 0.81); BGD19-C (GMQE = 0.93); BXK32-C (GMQE = 0.97).
(TIF)

S1 Table Surface plasmon resonance binding and fitting parameters. The calculated equilibrium dissociation constants, rate constants, and associated fitting statistics are provided for surface plasmon resonance binding experiments.
(DOCX)

S2 Table Complement assay IC50 data and non-linear regression fitting statistics. The calculated half maximal inhibitory concentration (IC50) values and associated fitting statistics are provided for each experimental set of complement functional assays.
(DOCX)
