# Supplementary figures and images for: Structural determination of the complement inhibitory domain of Borrelia burgdorferi BBK32 provides insight into classical pathway complement evasion by Lyme disease spirochetes
Source: PLoS Pathog. 2019 Mar 21;15(3):e1007659. doi: 10.1371/journal.ppat.1007659 (PMC6445466; doi:10.1371/journal.ppat.1007659)

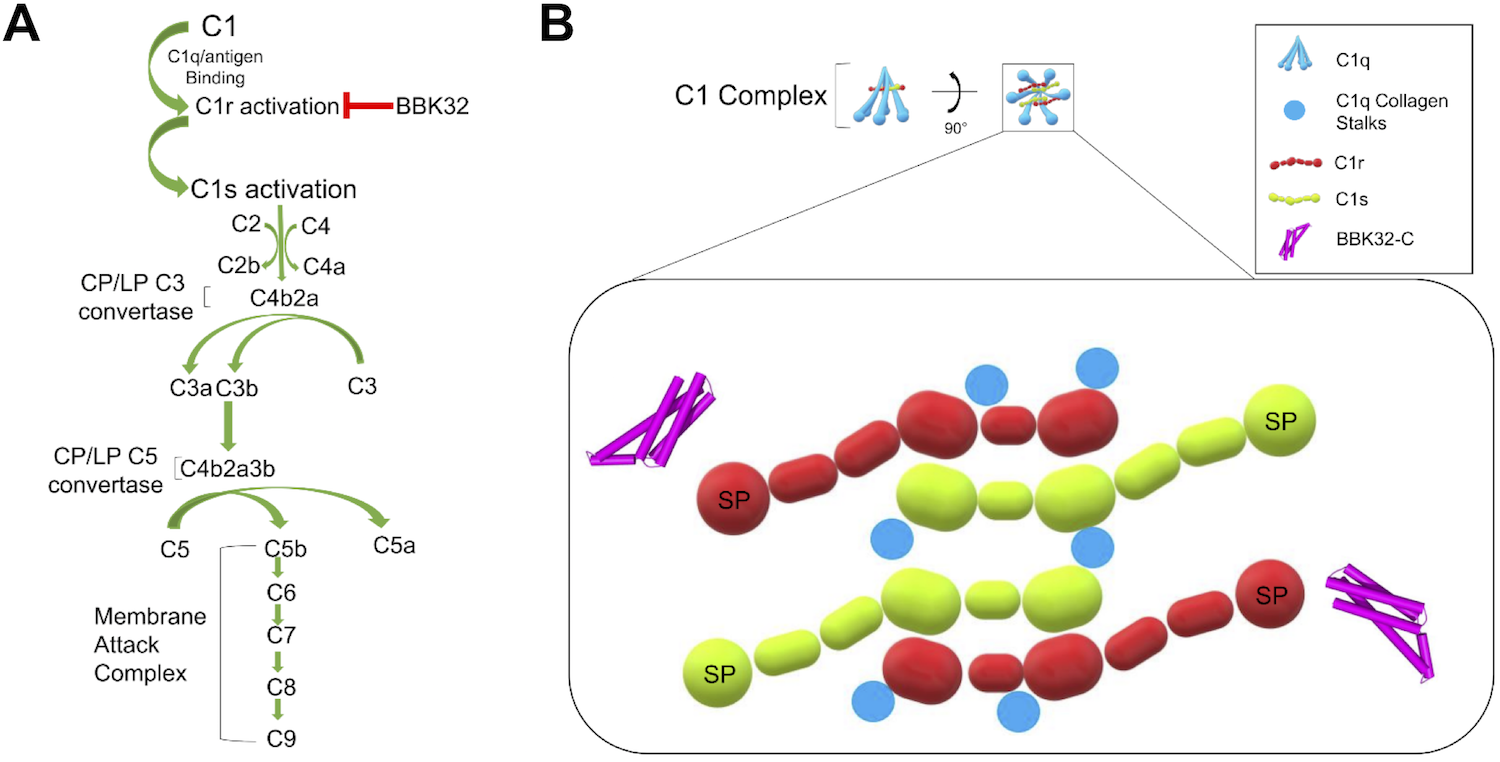

Supplement: S1 Fig — A) A schematic depiction of classical pathway complement activation is shown. C1q, the pattern recognition subunit of the C1 complex, binds to the targeted surface. C1q binding autoactivates the initiator serine protease, C1r, which then proteolytically cleaves C1s. Activated C1s cleaves complement proteins C2 and C4 leading to the surface formation of C4b2a, the CP (Classical Pathway)/LP (Lectin Pathway) C3 convertase. C3 convertases then cleave C3 into C3a and C3b leading to CP/LP C5 convertase formation (C4b2a3b). Cleavage of C5 by C5 convertases releases the anaphylatoxin C5a and leads the formation of the membrane attack complex (C5b-9) on the surface of the target cell. The membrane attack complex is a lytic pore structure that can directly kill the targeted cell(s). For Borrelia species, BBK32, or active orthologues of BBK32, can block activation of C1r and inhibit the classical complement cascade. B) A model for BBK32-mediated inhibition of the classical pathway. C1 complex, consists of C1q, which is composed of six collagen-like structures connected to six globular head domains. C1q binds a C1r2C1s2 heterotetramer to form C1 complex. The depiction of the arrangement of subunits within C1 is based on the work of Ugurlar and colleagues [87]. BBK32-C, binds the exposed serine protease (SP) domain of C1r and inhibits the autoproteolytic activation of C1r as well as the C1r-mediated cleavage of proC1s. Inhibition at this step halts the classical pathway at the initial proteolytic step and prevents formation of the downstream activation products of the cascade, including the membrane attack complex. (TIF) [file ppat.1007659.s001.tif]

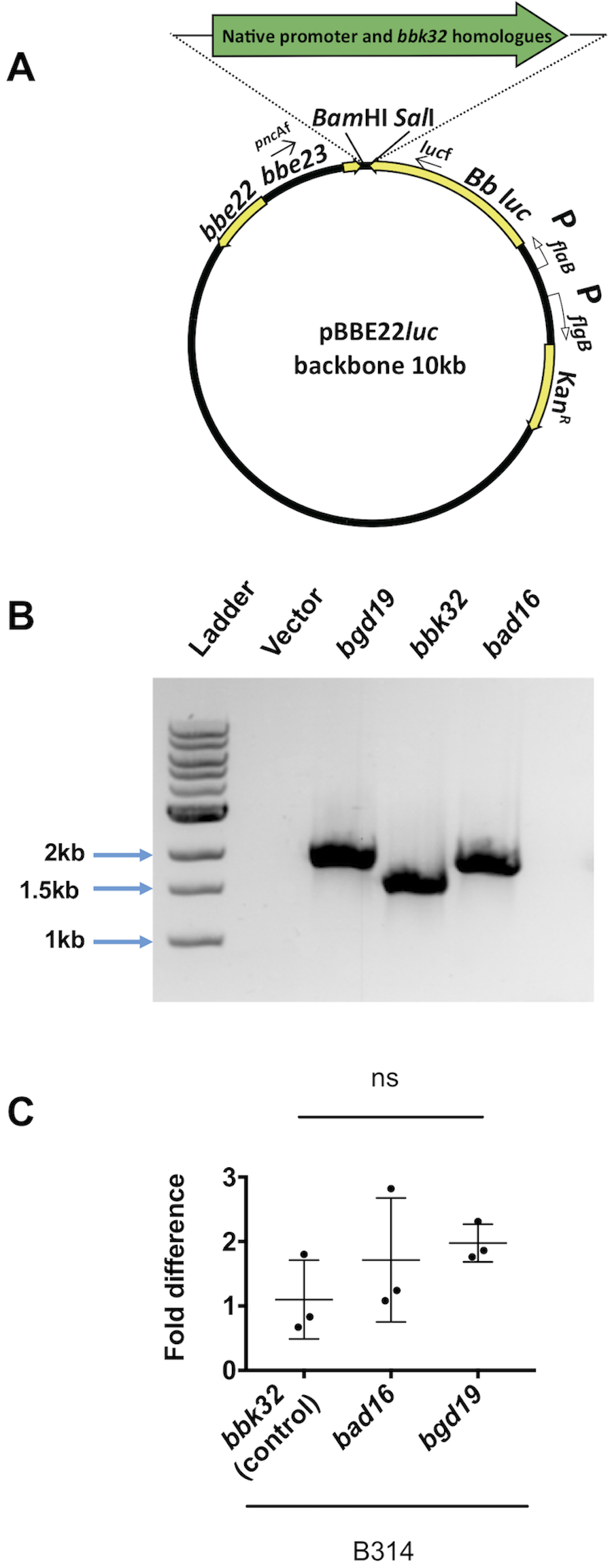

Supplement: S2 Fig — A) Schematic showing how the bbk32 orthologues bad16 and bgd19 from B. afzelii and B. garinii, respectively, were constructed using the pBBE22luc vector backbone. The resulting constructs were transformed into B. burgdorferi strain B314. B) PCR confirmation of bgd19 from B314/pBGD19, bbk32 from B314/pCD100, and bad16 from B314/pBAD16. All constructs contained the bad16, bbk32, bgd19 expressed from their native promoters. The Vector lane refers to the use of pBBE22luc as template for PCR with the oligonucleotide primers used to screen inserts. Values listed to the left indicate the size of markers in kilobases (kb). C) Quantitative RT-PCR shows that the expression of bbk32 orthologues (e.g., bad16 and bgd19) in strain B314 using their native promoters make transcripts equivalent or greater than B. burgdorferi sensu stricto bbk32. Expression of the bbk32 orthologues was compared relative to the constitutively expressed flaB gene (internal control). The qRT-PCR was done in triplicate and the mean values obtained for bbk32 was used as a comparator for the other orthologous genes (i.e., bad16 and bgd19). (TIF) [file ppat.1007659.s002.tif]

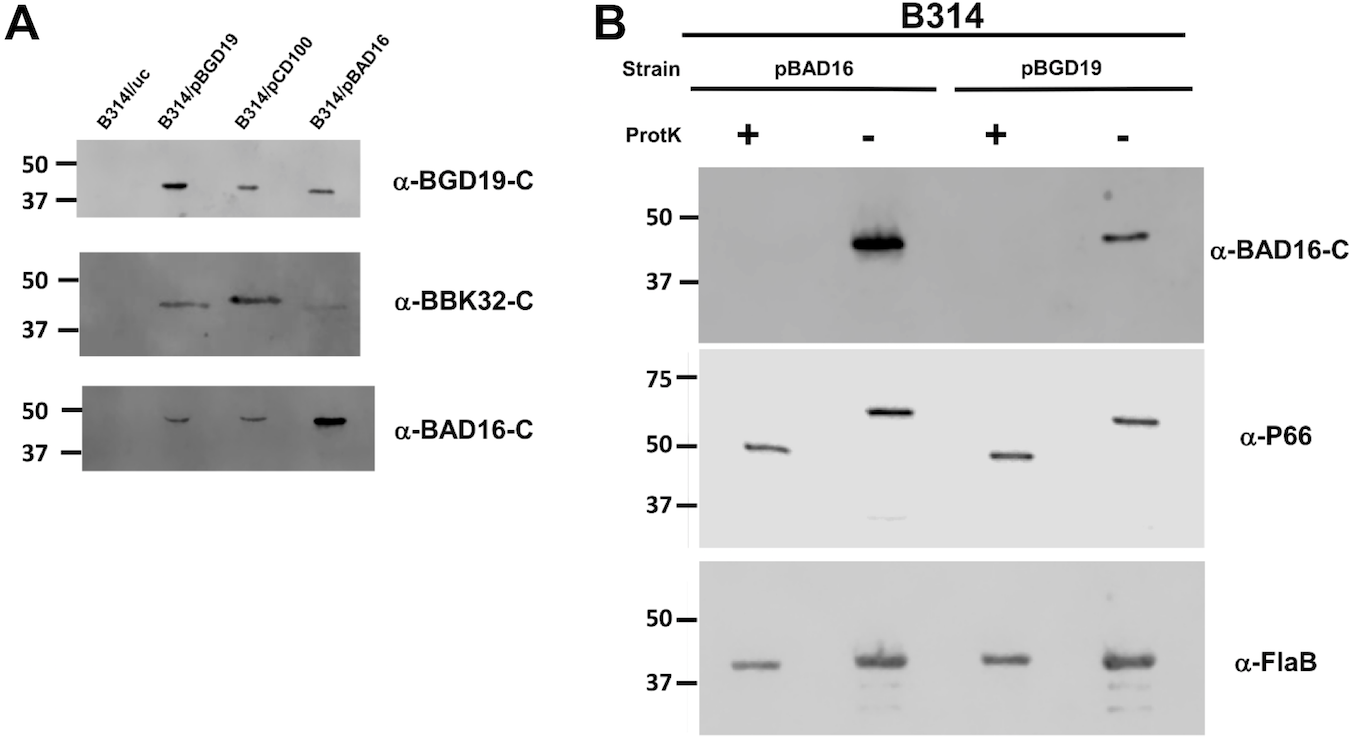

Supplement: S3 Fig — A) Antisera to BBK32 orthologues is cross reactive against all sensu lato isolates tested. Antisera against BGD19 from B. garinii, BBK32 from B. burgdorferi, and BAD16 from B. afzelii were tested in immunoblots of protein lysates from B. burgdorferi strain B314 containing the vector pBBE22luc (B314/luc), as well as B314 strain expressing B. garinii bgd19 (B314/pBGD19), B. burgdorferi bbk32 (B314/pCD100), and B. afzelii bad16 (B314/pBAD16). Individual membranes were then probed with rat polyclonal antisera against BGD19-C, BBK32-C, and BAD16-C as specified on the right. In all instances, the reagent used recognized its homologous target protein best but also showed significant reactivity to the other heterologous proteins. Markers in kDa are indicated on the left. B) The BBK32 orthologues encoded by B. afzelii and B. garinii, designated as BAD16 and BGD19, respectively, are surface exposed in the surrogate B. burgdorferi B314 strain. B314/pBAD16 and B314/pBGD19, encoding BAD16 and BGD19, respectively, were grown, washed, and then either resuspended with Proteinase K (ProtK; denoted with a “+”) or buffer alone (denoted with a “-“). Following processing, the resulting samples were subjected to SDS-PAGE and immunoblotted with antiserum directed against either BAD16-C, the outer membrane P66 protein, or the subsurface FlaB protein. Given the cross-reactivity of anti-BAD16 with all B. burgdorferi BBK32 orthologues (panel A), the fate of BGD19 could be assessed with the anti-BAD16-C reagent. Protein markers are indicted in the left (in kDa). (TIF) [file ppat.1007659.s003.tif]

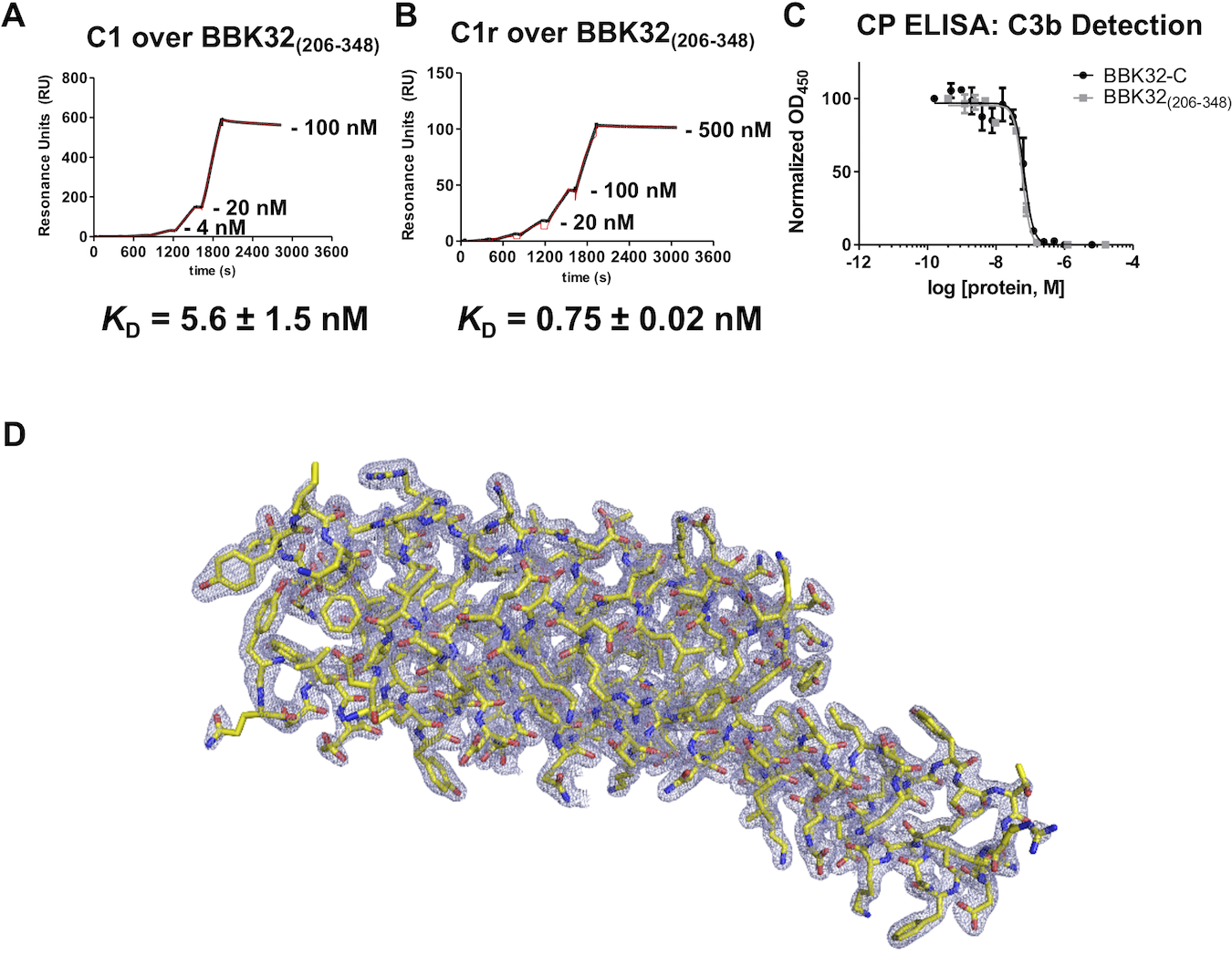

Supplement: S4 Fig — A-C) The construct used for crystallization, BBK32(206–348), which lacks six C-terminal residues relative to BBK32-C (i.e. BBK32206-354), retains high affinity C1r interaction and complement inhibitory properties. D) 2Fo-Fc density contoured at 1.2 σ for the entire BBK32 polypeptide. (TIF) [file ppat.1007659.s004.tif]

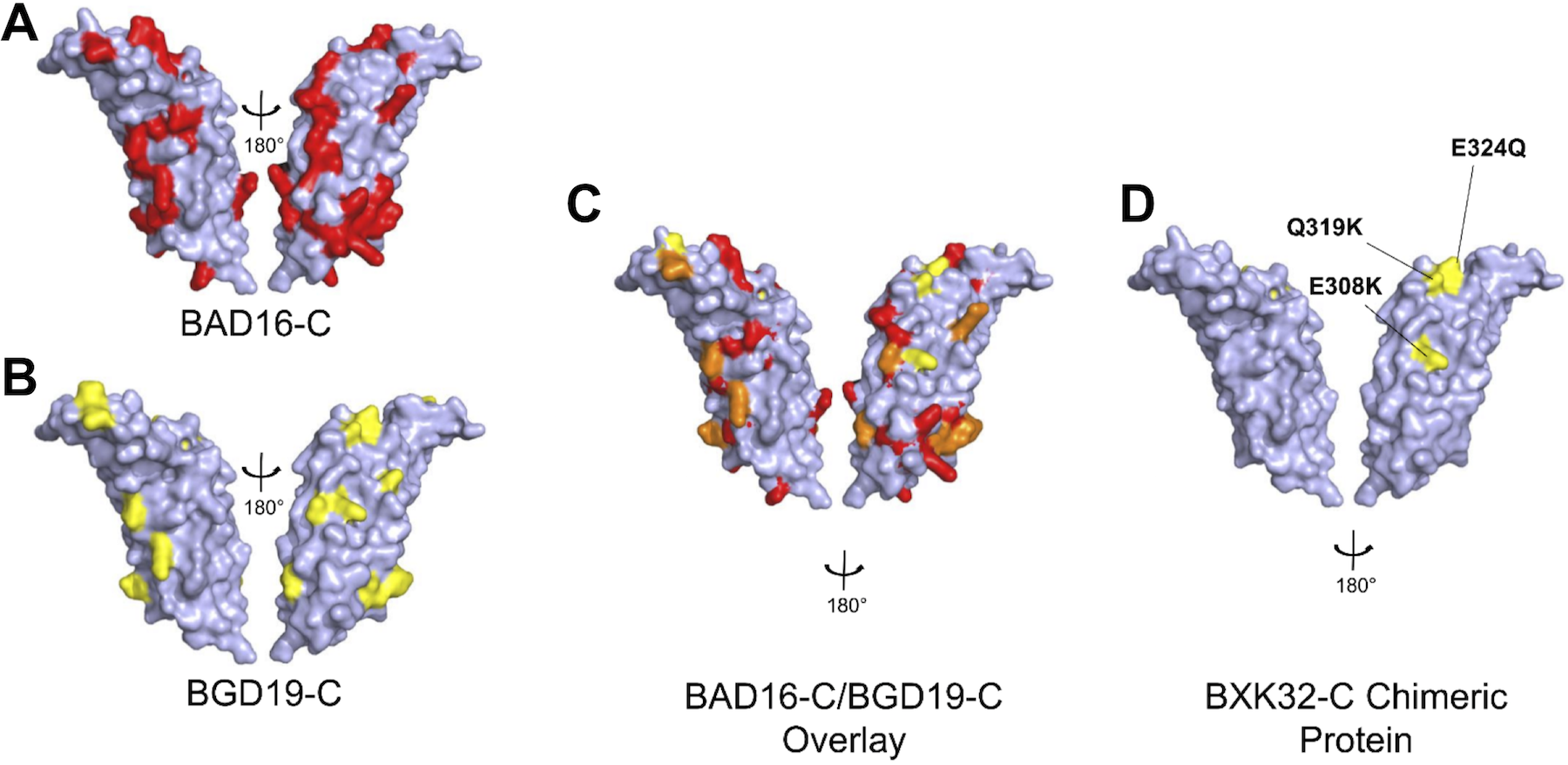

Supplement: S5 Fig — SWISS-MODEL was used to produce homology models of A) BAD16-C and B) BGD19-C that are based on the crystal structure of BBK32-C (PDB: 6N1L). Residues that are non-identical between BAD16-C and BBK32-C are shown in red on the protein surface (panel A), while residues that differ between BGD19-C and BBK32-C are shown in yellow (panel B). C) The homology models of BAD16-C and BGD19-C are structurally aligned. The coloring scheme shown in panels A/B is retained except overlapping residues are now colored in orange. Surfaces that remain yellow represent residues that are uniquely different in BGD19-C relative to BAD16-C. D) Three of these residues were selected for the BXK32-C chimera protein used in this study including residue positions 308, 319, and 324 (BBK32 numbering). A SWISS-MODEL homology model of the BXK32-C chimeric protein, also based on the BBK32-C crystal structure, predicts these three residues would remain solvent exposed. Global Model Quality Estimation (GMQE) is used by SWISS-MODEL to provide an estimate of model accuracy. Values range between 0 and 1, with higher numbers indicating higher model reliability and are as follows: BAD16-C (GMQE = 0.81); BGD19-C (GMQE = 0.93); BXK32-C (GMQE = 0.97). (TIF) [file ppat.1007659.s005.tif]
