# Supplementary material for: Structural determination of the complement inhibitory domain of Borrelia burgdorferi BBK32 provides insight into classical pathway complement evasion by Lyme disease spirochetes
Source: PLoS Pathog. 2019 Mar 21;15(3):e1007659. doi: 10.1371/journal.ppat.1007659 (PMC6445466; doi:10.1371/journal.ppat.1007659)
Supplement: S1 Table — The calculated equilibrium dissociation constants, rate constants, and associated fitting statistics are provided for surface plasmon resonance binding experiments. (DOCX) [file ppat.1007659.s006.docx]

**S1 Table. SPR: BBK32/C1 & C1r Binding and Fitting Parameters.**

| **Immobilized Ligand** | **Analyte** | ***K*_D_ ^a^**  **(nM)** | ***k*_a_**  **(M^-1^s^-1^)** | ***k*_d_**  **(s^-1^)** | ***χ*^2, b^** | ***K*_D_^c^**  **p-value** |
| --- | --- | --- | --- | --- | --- | --- |
| BBK32-C | C1 | 6.0 ± 0.60 | (8.4 ± 0.26) x 10^4^ | (5.0 ± 0.40) x 10^-4^ | 1.1, 1.6, 1.6 | ^d^ |
|  | C1r | 5.1 ± 1.3 | (1.7 ± 0.17) x 10^4^ | (8.6 ± 2.8) x 10^-5^ | 26, 17, 17 | ^d^ |
| BGD19-C | C1 | 3.0 ± 0.16 | (1.2 ± 0.02) x 10^5^ | (3.5 ± 0.21) x 10^-4^ | 10, 10, 12 | ** |
|  | C1r | 5.3 ± 0.31 | (1.5 ± 0.01) x 10^4^ | (8.1 ± 0.79) x 10^-5^ | 40, 29, 23 | ns |
| BAD16-C | C1 | 5.4 ± 1.1 | (1.1 ± 0.06) x 10^5^ | (6.2 ± 1.6) x 10^-4^ | 2.7, 2.7, 3.8 | ns |
|  | C1r | 2.2 ± 1.2 | (1.9 ± 0.17) x 10^4^ | (4.3 ± 2.6) x 10^-5^ | 35, 18, 22 | ** |
| BXK32-C | C1 | 4.7 ± 0.13 | (8.1 ± 0.19) x 10^4^ | (3.8 ± 0.09) x 10^-5^ | 3.4, 3.0, 4.1 | ns |
|  | C1r | 7.1 ± 1.0 | (2.2 ± 0.24) x 10^4^ | (1.6 ± 0.37) x 10^-4^ | 33, 25, 25 | ns |
| BBK32_(206-348)_ | C1 | 5.6 ± 1.5 | (1.0 ± 0.35) x 10^4^ | (5.4 ± 0.17) x 10^-4^ | 1.6, 14.3, 1.5 | ns |
|  | C1r | 0.75 ± 0.15 | (1.3 ± 0.01) x 10^4^ | (9.7 ± 0.66) x 10^-6^ | 2.4, 23, 6.3 | *** |
|  | C1r_auto_dig_ | 1.5 ± 0.20 | (1.7 ± 0.21) x 10^4^ | (2.6 ± 0.33) x 10^-5^ | 28, 300, 78 | ^e^ |
|  | rHIS-C1r-CCP2-SP | 3.9 ± 0.44 | (9.4 ± 0.58) x 10^3^ | (3.7 ± 0.27) x 10^-5^ | 0.3, 0.9, 0.8 | ^e^ |
|  | rHIS-C1r-CCP1 | NA | NA | NA | NA | ^e^ |
|  | rHIS-C1r-CCP1-CCP2 | NA | NA | NA | NA | ^e^ |

**^a^** All injection series were performed in triplicate and the mean value is reported for each as the *K*_D_ ± the standard deviation.

**^b^** A kinetic evaluation of sensorgrams was performed using T200 Evaluation Software where each dataset was fit to a one-to-one model of binding. Chi^2^ (*χ*^2^), which is a measure of the average deviation of the experimental data from the fitted curves, is provided for each of the three independent experiments conducted.

^c^ Graphpad Prism was used to perform an ordinary one-way ANOVA where calculated affinity constants (*K*_D_) where compared against the control analyte BBK32-C (Dunnett’s multiple comparison test). ***p* ≤ 0.01, ****p* ≤ 0.001. ns, not significant.

^d^ This is the data used as the reference for all statistical comparisons below with other proteins tested

^e^ Unique analyte utilized; no comparison was made using the analyte molecules listed.
