# Supplementary material for: Structural determination of the complement inhibitory domain of Borrelia burgdorferi BBK32 provides insight into classical pathway complement evasion by Lyme disease spirochetes
Source: PLoS Pathog. 2019 Mar 21;15(3):e1007659. doi: 10.1371/journal.ppat.1007659 (PMC6445466; doi:10.1371/journal.ppat.1007659)
Supplement: S2 Table — The calculated half maximal inhibitory concentration (IC50) values and associated fitting statistics are provided for each experimental set of complement functional assays. (DOCX) [file ppat.1007659.s007.docx]

**S2 Table. Complement Assay IC_50_ Data and Non-linear Regression Fitting Statistics**

| **Protein** | **Assay** | **IC50 (nM)** | **95% confidence interval (nM)** | **R^2^** |
| --- | --- | --- | --- | --- |
| BBK32-C | ELISA:C3b | 14 | 14 to 15 | 0.9938 |
|  | ELISA: MAC | 13 | 11 to 15 | 0.9707 |
|  | Hemolysis | 170 | 130 to 220 | 0.8816 |
| BGD19-C | ELISA:C3b | 32 | 29 to 36 | 0.9875 |
|  | ELISA: MAC | 35 | 31 to 39 | 0.9604 |
|  | Hemolysis | 7,600 | 2,400 to 165,000 | 0.5751 |
| BAD16-C | ELISA:C3b | 9.6 | 8.8 to 10 | 0.9885 |
|  | ELISA: MAC | 5.9 | 5.5 to 6.5 | 0.9864 |
|  | Hemolysis | 55 | 49 to 59 | 0.944 |
| BXK32-C | ELISA:C3b | 32 | 30 to 33 | 0.997 |
|  | ELISA: MAC | 18 | 17 to 20 | 0.9912 |
|  | Hemolysis | n.d. |  |  |
